# Genome-wide analysis of DWD proteins in soybean (*Glycine max*): Significance of Gm08DWD and GmMYB176 interaction in isoflavonoid biosynthesis

**DOI:** 10.1371/journal.pone.0178947

**Published:** 2017-06-06

**Authors:** Shaomin Bian, Xuyan Li, Hemanta Mainali, Ling Chen, Sangeeta Dhaubhadel

**Affiliations:** 1 College of Plant Science, Jilin University, Changchun, Jilin, China; 2 Agriculture and Agri-Food Canada, London Research and Development Centre, London, ON, Canada; 3 Department of Biology, Western University, London, ON, Canada; Institute of Genetics and Developmental Biology Chinese Academy of Sciences, CHINA

## Abstract

A subset of WD40 proteins with DWD motif has been proposed to serve as substrate receptor of DDB-CUL4-ROC1 complex, thereby getting involved in protein degradation via ubiquitination pathway. Here, we identified a total of 161 potential DWD proteins in soybean (*Glycine max*) by searching DWD motif against the genome-wide WD40 repeats, and classified them into 20 groups on the basis of their functional domains and annotations. These putative *DWD* genes in soybean displayed tissue-specific expression patterns, and their genome localization and analysis of evolutionary relationship identified 48 duplicated gene pairs within 161 *GmDWD*s. Among the 161 soybean DWD proteins, Gm08DWD was previously found to interact with an isoflavonoid regulator, GmMYB176. Therefore, Gm08DWD and its homologue Gm05DWD were further investigated. Expression profile of both genes in different soybean tissues revealed that *Gm08DWD* was expressed higher in embryo, while *Gm05DWD* exhibited maximum transcript accumulation in leaf. Our protein-protein interaction studies demonstrated that Gm08DWD interacts with GmMYB176. Although Gm08DWD was localized both in nucleus and cytoplasm, the resulting complex of Gm08DWD and GmMYB176 was mainly observed in the nucleus. This finding is consistent with the functional localization of CUL4-E3 ligase complex. In conclusion, the survey on soybean potential DWD protein is useful reference for the further functional investigation of their DDB1-binding ability. Based on the functional investigation of Gm08DWD, we speculate that protein-protein interaction between Gm08DWD and GmMYB176 may lead to the degradation of GmMYB176 through CUL4-DDB1complex.

## Introduction

The WD40 domain containing proteins are highly conserved and are found as tandem repeat units across a wide variety of eukaryotic organisms. They are also called as WD40-repeat containing (WDR) proteins. Each WD40 domain comprises of approximately 40 amino acid central region containing a glycine-histidine (GH) dipeptide at the N-terminal and the conserved tryptophan-aspartate (WD) dipeptide at the C-terminal end [1]. The two dipeptides of WD40 domain are separated by a region of variable lengths that exhibits low level of sequence conservation [2]. WDR proteins function as scaffolding proteins for assembly of larger complexes thus mediating diverse protein-protein and protein-DNA interactions [3].

A subgroup of WDR proteins contain a conserved motif within the WDR named as WDxR or DWD box. The DWD box within the WDR binds to DAMAGED DNA BINDING1 (DDB1) proteins that serve as substrate receptors for CUL4-based E3 ubiquitin ligase complex [4–6]. The DWD proteins recognize a variety of substrates, thereby dictating the specificity of the ubiquitination process for protein degradation in different organisms [5]. As revealed by the structure-based sequence analysis, majority of the DWD proteins possess either one or two WDxR motif (s) located within the WDR domain [5, 6]. The WDxR motif consists of 16 amino acids, of which 4 are highly conserved: Asp (or Glu) 7, Trp (or Tyr) 13, Asp (or Glu) 14, and Arg (or Lys) 16 (D/E-W/Y-D/E-R/K). The conserved Asp and Arg residues within the WDxR motif play a critical role for the interaction between DWD proteins and DDB1 [4]. The amino acid residue Arg-16 of the first DWD motif is exposed on the surface of the bottom face of the β propeller thus allowing the accessibility for the interaction with DDB1 [7]. Therefore, WDxR motif is considered as the signature motif of potential substrate receptors of CUL4-DDB1 ligase complex.

Generally, DWD proteins exist in all eukaryotes [5]. They function in regulating a wide range of cellular processes, including cell proliferation, light-dependent growth regulation, modulation of chromosomal structure, DNA repair, and genomic integrity by ubiquitinating key regulators [8–10]. Therefore, significant efforts have been made to identify DWD proteins in various plant species. Recently, the availability of the whole genome sequence has greatly contributed to genome-wide identification of DWD proteins in many plant species. For example, Arabidopsis genome contains at least 269 putative WDR proteins [11] whereas foxtail millet [12] and rice [13] genomes encode 225 and 200 WDRs, respectively. Among these WDR proteins, 85 and 78 are putative DWD proteins in Arabidopsis and rice, respectively [14] whereas out of 269 putative WDR proteins, 100 are DWDs in tomato [15]. A total of 27 Arabidopsis DWD proteins interact with DDB1 protein [9, 16–18]. These studies have provided insights into the biological role of many of the DWD proteins. For example, Arabidopsis CUL4-DDB1 interacts with COP1-SPA complex to regulate photomorphogenesis and flowering time [19]. MSI4 interacts with CUL4–DDB1 and a PRC2-like complex to control epigenetic regulation of flowering time in Arabidopsis [20]. Arabidopsis CUL4–DDB1 complex also interacts with MSI1 to maintain MEDEA parental imprinting [21]. DWA1, DWA2 and DWA3, as components of CUL4-based E3 ligases, act as negative regulators in ABA signal transduction, and it has been proposed that DWA1 and DWA2 are substrate receptors of CUL4-DDB1 for marking ABI5 degradation [22, 23]. PRL1 was shown to be associated with the CUL4 complex and responsible for the degradation of AKIN10 (Arabidopsis SNF1 Kinase Homolog 10) [14, 24]. WDR55 has been shown to interact with DDB1 and that this interaction was required for gametogenesis and seed development [17]. Most recently, it has been reported that ASG2 can act as negative regulator to respond ABA via its interaction with DDB1 in Arabidopsis [18].

Soybean (*Glycine max* [L.] Merr.) is one of the important grain legumes for seed protein and oil, and a predominant source of isoflavonoids and saponins. Isoflavonoids are legume-specific plant natural compounds that play important function as signal molecules in nitrogen fixation [25, 26] as well as biotic and abiotic stresses [27, 28]. Many clinical studies have suggested a role for isoflavonoids in human health and nutrition [29]. We have identified an R1 MYB transcription factor GmMYB176 that regulates *CHS8* gene expression and isoflavonoid biosynthesis in soybean [30]. GmMYB176 was found to interact with a DWD protein (Gm08DWD) in a yeast two hybrid (Y2H) screening where GmMYB176 was used as a bait protein and the soybean embryo proteins (50 and 60 days after pollination) as preys. In this study, we provide a genome-wide characterization of DWD proteins in soybean, and present a detailed characterization of Gm08DWD that interact with isoflavonoid regulator GmMYB176.

## Materials and methods

### Plant materials

Soybean cultivar Harosoy63 seeds were planted at Agriculture and Agri-Food Canada experimental station in London following regular agronomic practices. Soybean tissues were collected from 5–10 random plants, frozen in liquid nitrogen and stored at -80°C. *Nicotiana benthamiana* plants were grown in pots under 16 h light at 25°C and 8 h dark at 20°C with 70–80% relative humidity. Arabidopsis seeds were incubated at 4°C in the dark for 3 days before moving them into the growth room. Plants were grown for 16 h in the light at 22°C/8 h in the dark at 18°C.

### *In silico* analysis

The putative GmWD40 proteins were identified by the key word search ‘WD40’ against Phytozome 12 *Glycine max* Wm82.a2.v1 database (www.phytozome.net). This search used 201 ontologies from multiple databases including Pfam, Panther, KOG, EC, GO, KEGG Orthology and Cluster KEGG Orthology. Each candidate protein identified from Phytozome soybean database was confirmed for their WD40 domain annotation and architecture by using Simple Modular Architecture Research Tool (SMART) database available at http://smart.embl.de [31]. Following the methods used by Lee et al. [14] and Zhu et al. [15], we first confirmed for the presence of WD40 repeat in each WDR protein using SMART, and then DWD motif was manually identified by using the conserved amino acid sequence ([IFVL]-[IFVL]-[AGST]-[AGST]-[AGST]-x-[DE]-x(2)-[IFVL]-x-[IFVL]-[WY]-[DE]-[IFVL]-[RK]) search against each WD40 repeat sequence. Additional conserved motifs or domains other than DWD were identified using the SMART database.

### Multiple sequence alignment and phylogenetic analysis

The amino acid sequences of GmDWD proteins were aligned using ClustalW and the alignment was imported to MEGA7 for phylogenetic analysis. The neighbor-joining method was used to generate a phylogenetic tree based on the midpoint and the p-distance model [32]. To assess statistical significance of the phylogenetic trees, interior branch tests were conducted using 1000 replicates.

### Chromosomal localization and gene duplication

The gene location and chromosome number for each of the putative *GmDWD* was obtained from Phytozome v11.0.7 database (www.phytozome.net). Based on their chromosomal locations *GmDWD*s were mapped manually on the chromosomes.

As described in detail previously [33],*GmDWD*s in duplicated genomic regions and Ka/Ks values for each duplicated *GmDWD*s were obtained for syntenic mapping from batch download option of Plant Genome Duplication Database (http://chibba.agtec.uga.edu/). Segmental duplication was defined as the homologous genes located on duplicated chromosomal blocks, while two paralogs with less than 5 gene loci in-between were set as tandem duplication.

### Gene expression analysis

The fragments per kilobase of transcript per million mapped reads (FPKM) values for each *GmDWD* were retrieved to assess the transcript level of *GmDWD*s by tracking nine tissue gene-level expression from Phytozome database (http://www.phytozome.net), and the data were normalized across tissues. The heatmap for *GmDWD* genes was produced using R’sheatmap2 function from the gplots CRAN library (http://CRAN.R-project.org/package=gplots).

For quantitative RT-PCR (qPCR) and RT-PCR analysis, total RNA was isolated according to Wang and Vodkin [34] from root, stem, leaf, flower buds, flower, embryo (30, 40, 50 days after pollination), pod wall and seed coat tissues collected from soybean plants. First strandcDNA was synthesized using a Quantitect Reverse Transcription kit (Qiagen Inc.). qPCR was performed using a CFX96 real-time PCR detection system (Bio-Rad Inc.) and QuantiTect SYBR Green PCR kit (Qiagen Inc.). The data were analyzed using a CFX manager, and *Soybean ubiquitin-3* (*SUBI3*) was set as an internal reference for data normalization [35, 36]. The experiment was conducted with three technical replicates for each independent biological replicate. The primer sequences are listed in S1 Table.

### Histochemical GUS assay

Upstream regions from translation start site (~1.28 kb) for *Gm05DWD* and *Gm08DWD* were cloned into pMDC162 vector using the primers listed in S1 Table, and then transferred into Arabidopsis using *Agrobacterium tumefaciens* mediated floral dip method [37]. For GUS staining, seedlings and different tissues were immersed in GUS staining solution (0.5 mg/mL 5-bromo-4-chloro-3-indolyl-glucuronide, 20% methanol, 0.01 M Tris-HCl, pH 7.0) with vacuum for 15 min, and then incubated at 37°C overnight [38]. The samples were cleared by sequential incubation in 75% and 95% ethanol.

### Targeted yeast two hybrid assay

For the targeted Y2H assay, full-length cDNA of *GmMYB176* and *Gm08DWD* were PCR amplified using gene-specific primers (S1 Table) and cloned into the Gateway entry vector pDONORZeo by homologous recombination to obtain pDONZ-*GmMYB176* and pDONZ-*Gm08DWD* and sequences were confirmed. The entry constructs were recombined into the Y2H destination vectors pGBKT7-DEST (bait) and pGADT7-DEST (prey) to obtain pGBKT7-*GmMYB176*, pGBKT7-*Gm08DWD*, pGADT7-G*mMYB176* and pGADT7-*Gm08DWD*. As described in detail previously [33], the vectors in different combinations were co-transformed into yeast strain AH109 and selected on SD/-Leu/-Trp agar plates. Selected individual yeast transformants were grown in liquid medium and 5 μL AH109 culture with a series of 10-fold dilution was dropped onto SD/-Leu/-Trp and SD/-Ade/-His/-Leu/-Trp plates and grown for 8 days at 30°C. Empty vectors were used as negative controls.

### Subcellular localization and bimolecular fluorescence complementation (BiFC) assay

For BiFC assay, *Gm08DWD* full-length cDNA was introduced to the BiFC vectors pEarlyGate201-YN or pEarlyGate202-YC (pEG201-YN or pEG202-YN) [39]. Construction of BiFC vectors containing *GmMYB176* was described previously [40]. To determine the subcellular localization of Gm08DWD, the vector pEarlyGate101 was used to generate a translational fusion of Gm08DWD with YFP [41]. For BiFC assay, *A*. *tumefaciens* culture harboring pEG201-*Gm08DWD-YN* and pEG202-*GmMYB176-YC* or pEG201-*GmMYB176-YN* and pEG201-*Gm08DWD-YC* were mixed 1:1 and co-transformed into tobacco leaf epidermal cells by infiltration as described in detail previously [33]. The YFP was observed by a Leica confocal microscope (http://www.leica.com/). For subcellular localization, pEG101-*Gm08DWD* was transiently expressed in tobacco epidermal cells, followed by confocal microscopy as described above.

## Results

### Identification and classification of GmDWD proteins in soybean

**Fig 1:**
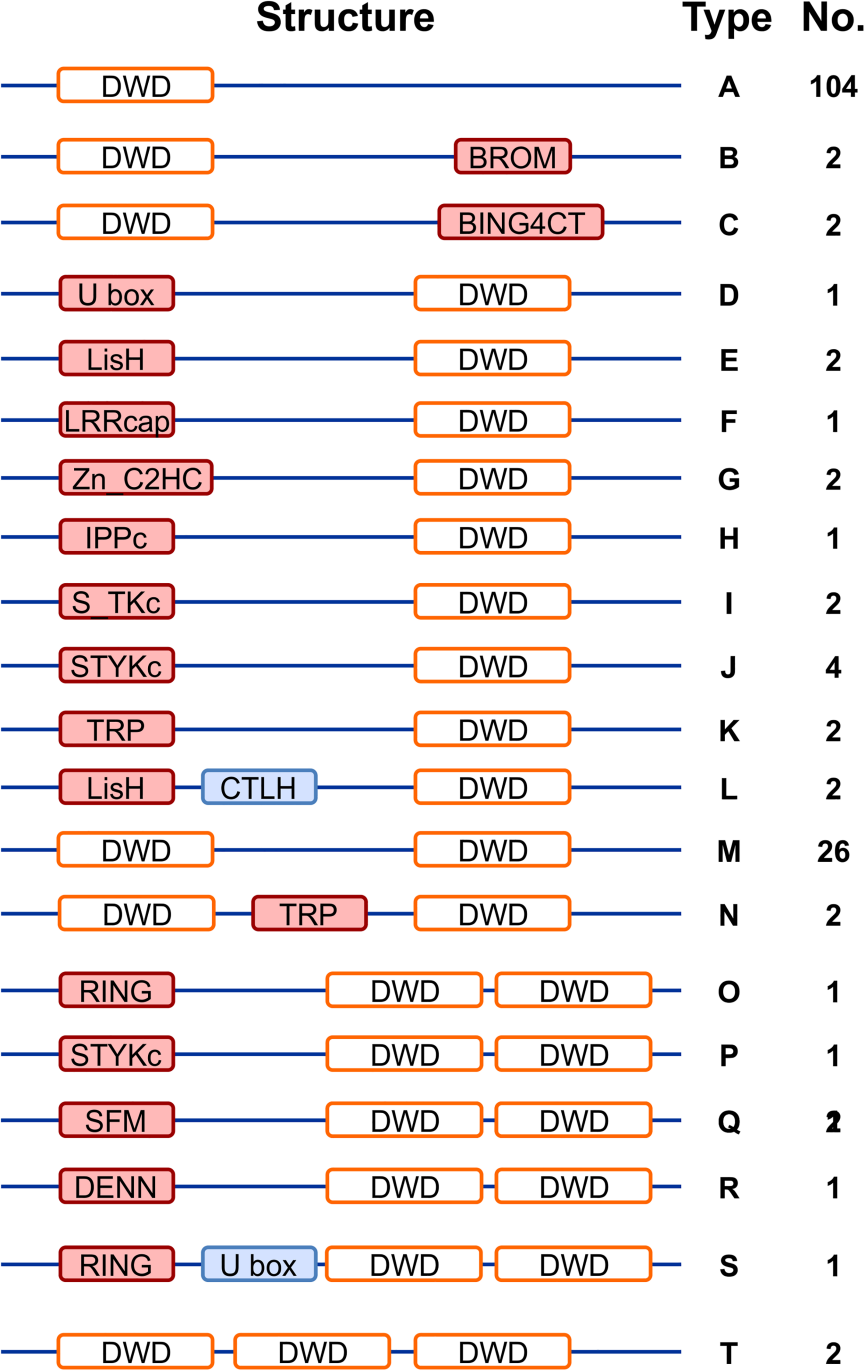
Structure of candidate GmDWD proteins and their domain diversity in soybean. The candidate GmDWD proteins are categorized into 20 types (A to T). Number of candidate GmDWD proteins belonging to each type is indicated.

Since DWD motifs are found within WD40 repeats, we first retrieved all annotated WD40 proteins from Phytozome 12 *Glycine max* Wm82.a2.v1 using key word search. This search identified a total of 471 putative WD40 proteins in soybean genome. Subsequently, WD40 regions within each predicted WD40 protein were manually searched for the 16 conserved amino acid sequence for DWD motif: [IFVL]-[IFVL]-[AGST]-[AGST]-[AGST]-x-[DE]-x(2)-[IFVL]-x-[IFVL]-[WY]-[DE]-[IFVL]-[RK]. A total of 161 putative DWD proteins were identified by this search in soybean (S2 and S3 Tables). Among them, 125 proteins contained single DWD domain, 34 contained 2 domains, and 2 proteins contained 3 DWD domains (Fig 1). A search for soybean DWD orthologs in Arabidopsis [14] found 130 DWD proteins, where their identity ranged from 43.6% to 89.1% (S2 Table). Among them, 62 GmDWDs corresponded to the DDB1 interactors in Arabidopsis that are experimentally validated [9, 16–18]. These results suggest that these DWD proteins are conserved in both legume and non-legume plants.

The candidate DWD proteins were further assessed for additional domains using SMART analysis. Based on the number of DWD motif and domain diversity, the 161 GmDWD proteins were classified into 20 types ranging from A to T (Fig 1). Majority of GmDWD proteins (132) contained either single or multiple DWD domain (s) whereas some GmDWDs (29) contained other known functional domains (Fig 1 and S2 Table). For example, Glyma.09G037300 and Glyma.15G142600 include BROMO domain that function in binding with acetyl-lysine during histone acetylation [42]. Similarly, 4 predicted DWD proteins contain a LisH motif that helps to regulate microtubule dynamics by assisting microtubule dimerization [43]. Some of other DWDs with additional domains are as following (Fig 1 and S2 Table): the TPR domain that facilitates protein-protein interaction was found in 2 DWD proteins. The E2-dependent ubiquitination-related domains were detected in 3 DWD proteins. Glyma.08G040500 contains the DENN domain implicated in the regulation of mitogen-activated protein kinase signaling pathways [44]. Glyma.20G131200 contains LRRcap facilitating the interaction with U2 snRNA. Five DWD proteins have the STYKc domain that possesses catalytic specificity for tyrosine kinase whereas two DWDs contain S_TKc domain that have serine/threonine protein kinase catalytic activity. The DWDs with BING4CT domain at the C- terminus end may function as nucleolar WDR proteins. Glyma.02G226300 and Glyma.14G193200 have ZnF_C2HC domain. These additional domains might be implicated in the recognition of a variety of substrates since the DWD motif binds DDB1 while other portions of the protein may bind substrates. On the basis of the functional annotation, we grouped the 161 GmDWD proteins into 8 categories (S1 Fig), where majority of DWDs are predicted to involve in RNA processing (22.4%) and signal transduction (23.0%).

### Chromosomal localization and phylogenetic analysis of soybean *DWD* genes

**Fig 2:**
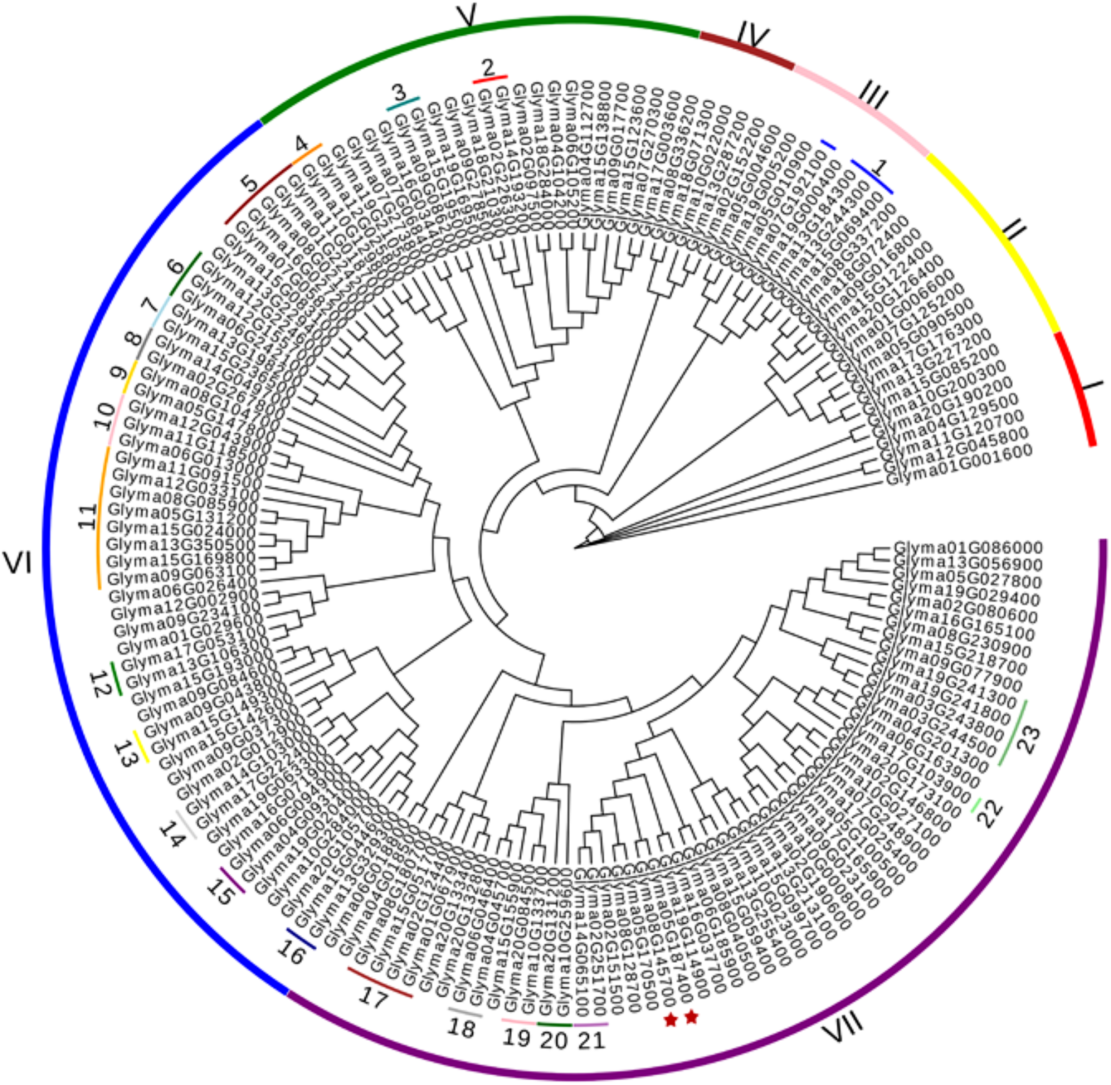
Phylogenetic relationships of candidate DWD proteins in soybean. A neighbor-joining tree was generated by MEGA7 software using putative amino acid sequences of 161 GmDWDs. Clusters are indicated by roman letters (I-VII). Candidate GmDWDs, whose Arabidopsis orthologs are functionally characterized are indicated with numbers: 1, DWA3 (OAP14017); 2, DDB2 (Q6NQ88); 3 DWD motif protein 1 (AAN28840); 4, WDR55 (BAC42145); 5, SPA1-2(OAP08846, Q9T014); 6, SPA3 (AEE75647); 7, FY (OAO90078); 8, COP1 (P43254); 9, HTD1 (AEC06893); 10, AGB1 (P49177); 11, MSI1 (AAL24356); 12, DWA1 (AEC06880); 13, PRL1 (CAB78632); 14, ULCS1 (AAO00945); 15, ASG2 (AAN12885); 16, DWD-motif protein 2 (NM_001203085); 17, RAE1 (ABF18994); 18, CSA-1 (OAP13038); 19, MAC3B (AEC08817); 20, DHU1 (AAO64896); 21, DWD-motif protein 3 (AAU94425); 22, DRS1 (AEE36439); 23, LRS1 (ANM63427). Gm05DWD and Gm08DWD are marked with asterisks.

To explore the evolutionary relationships of 161 predicted DWDs in soybean, a phylogenetic tree was generated. The results revealed that the putative DWD proteins could be categorized divided into 7 distinct groups (I to VII) (Fig 2). The groups VI and VII contained 51 and 57 candidate GmDWDs, respectively, while relatively fewer GmDWDs clustered in group I-V. It was observed that GmDWDs with the same or similar annotations were clustered together in the phylogenetic tree. For example, 8 CAF1/NURF55/MSI1 proteins clustered together with each other in the group VI including Glyma.05G131200, Glyma.08G085900, Glyma.09G063100, Glyma.11G091500, Glyma.12G033100, Glyma.13G350500, Glyma.15G024000 and Glyma.15G169800, while 4 mRNA export protein (Glyma.01G067900, Glyma.02G124400, Glyma.08G180700 and Glyma.15G051700) formed a clade in the group VII. Furthermore, 62 GmDWDs, whose orthologs in Arabidopsis showed interaction with CUL4-based E3 ligase complex such as DDB2, SPA1-3, COP1, MSI1-4, REA, ASG2, ULCS1, PRL1, DWA1-3, CSA-1, DHU1, MAC3B, LRS1, HTD1, AGB1, DRS1, clustered in groups III, V, VI and VII (Fig 2 and S2 Table). It has been proposed that COP1 forms multiple complexes with SUPPRESSOR OF PHYA-105 (SPA1-4) family members to suppress photomorphogenic growth [19]. In the phylogenetic tree, the GmDWDs orthologs of SPAs, COP1, MSI and FY in Arabidopsis were clustered into a discrete clade (Fig 2), suggesting that they have close relationship during evolution.

**Fig 3:**
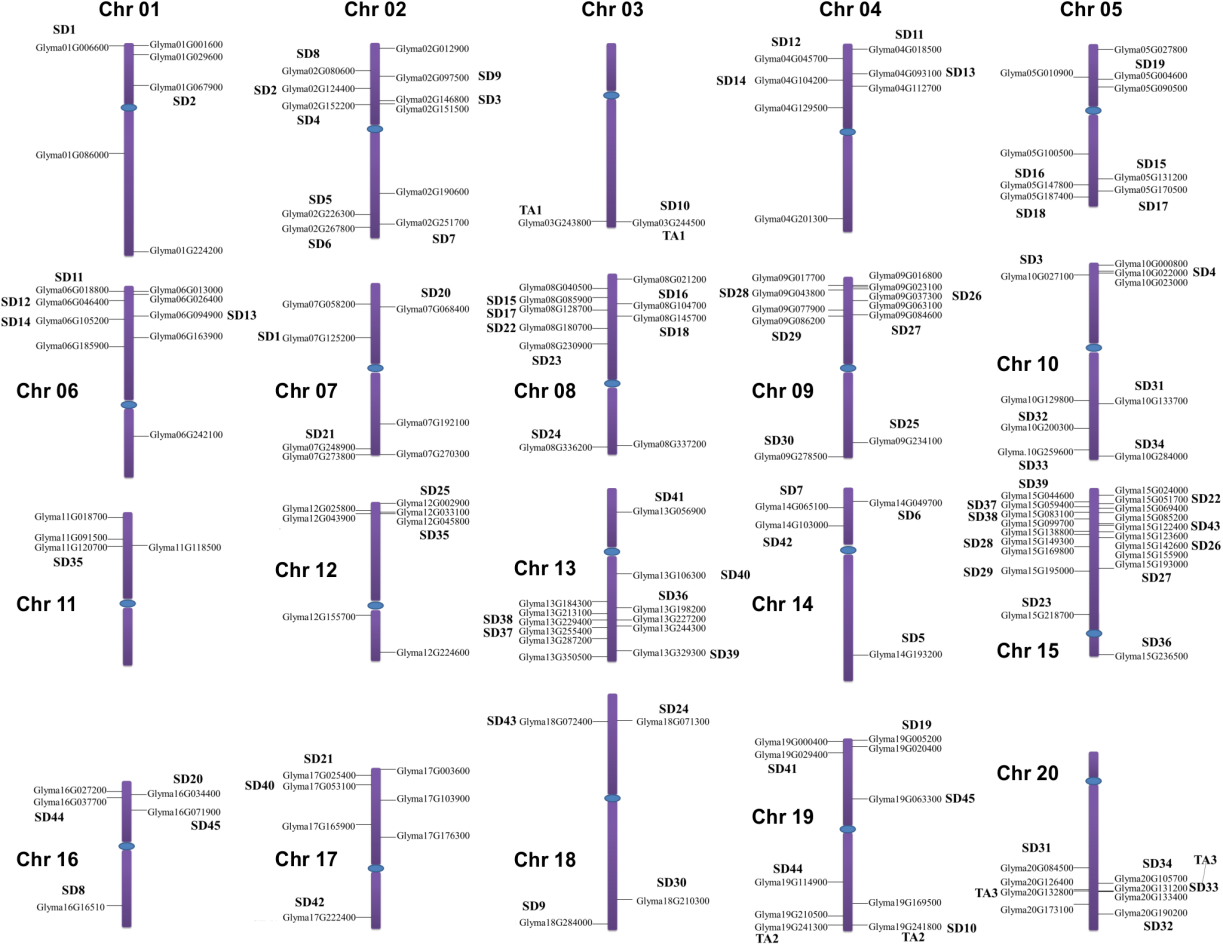
Chromosomal localization and duplicated *GmDWD* gene pairs in soybean. Segmental duplications (SD) are indicated by SD1-SD45, and tandem amplifications (TA) are indicated by TA1-TA3. Chromosomes are drawn to scale and chromosome numbers are shown beside each chromosome. Centromeres are indicated by blue ovals.

The genomic distributions of all candidate GmDWD genes were determined by their location on soybean chromosome. The *GmDWD* genes were dispersed unevenly on all 20 chromosomes in soybean (Fig 3). Some chromosomes contain a relatively high number of *GmDWD* genes while others contain very few. For example, 19 *DWD* genes are present on chromosome 15, followed by 12 genes on chromosome 13 whereas only 2 *GmDWD* genes are present on chromosome 3 (Fig 3). Segmental duplication and tandem amplification of chromosomal regions are main contributors for gene extension during evolution [45]. Generally, tandem amplification is defined as two paralogs separated by less than five genes in the same chromosome [46]. Three pairs of *GmDWD* genes in the same chromosome are found close to each other such as Glyma.03G243800 and Glyma.03G244500; Glyma.19G241300 and Glyma.19G241800; Glyma.20G132800 and Glyma.20G133400 (Fig 3 and S2 Table). These genes share greater than 93.3% of sequence identity at both protein and nucleotide level, suggesting that they are likely derived from tandem amplification of chromosomal regions. Based on coordinates of *GmDWD* genes, we further investigated whether traceable genome duplications contributed to the expansion of *GmDWD* genes in soybean. As indicated in S4 Table, 45 sets of *GmDWDs* were mapped on 34 distinct duplicate blocks, and each set of *GmDWD*s were clustered into a discrete clade in phylogenetic tree with 75.5–99% sequence identity, suggesting that these pairs of *GmDWD*s on the same block are possibly derived from segmental duplication events during evolution. To investigate the selective evolutionary pressure on *GmDWD* gene divergence after duplication, the non-synonymous/synonymous substitution ratio (Ka/Ks) was retrieved for the 46 duplicated pairs of *GmDWD* genes from Plant Genome Duplication Database. As shown in S4 Table, the Ka/Ks value of all the duplicated gene pairs ranged from 0.025 to 0.574. Since the Ka/Ks values were less than 1, these genes might have undergone a purifying selection with limited functional divergence after duplication.

### Soybean *DWD* genes display tissue-specific expression pattern

**Fig 4:**
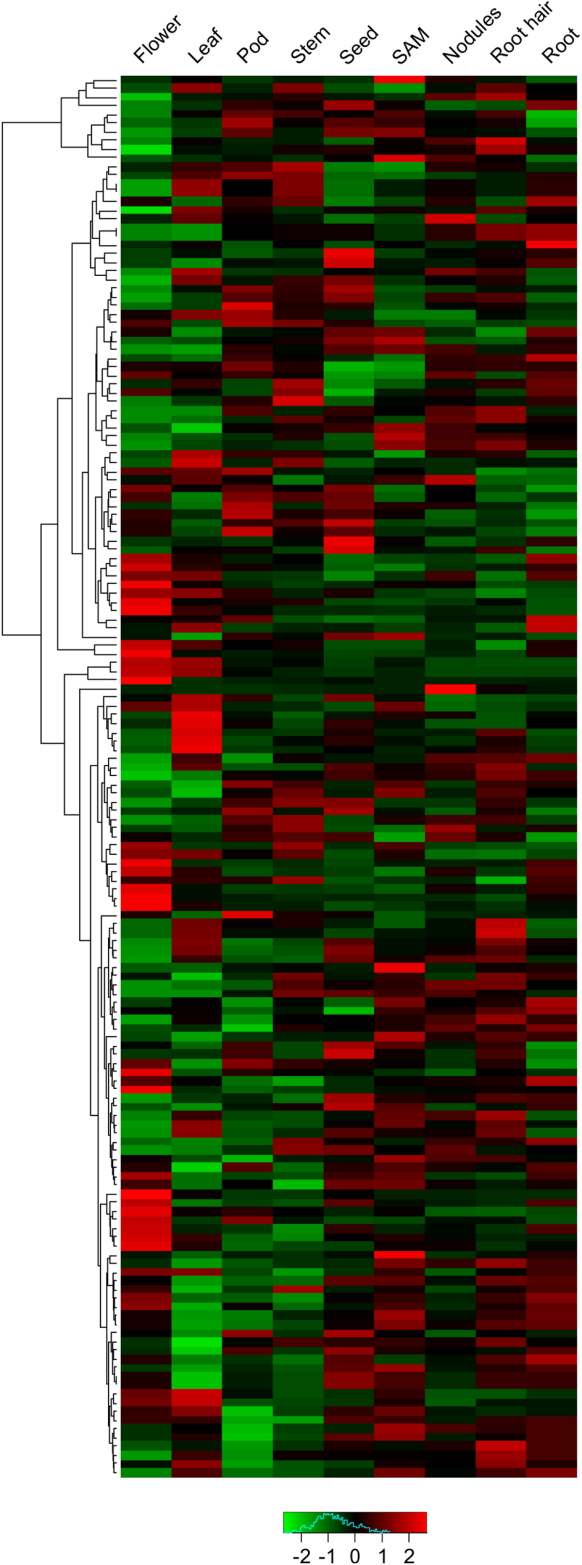
Expression analysis of soybean *DWD* genes. The transcript profiling data of soybean tissues were extracted from Phytozome database (http://www.phytozome.net) for heatmap generation. The color scale above the heat map indicates gene expression levels, low transcript abundance indicated by green color and high transcript abundance indicated by red color. SAM, shoot apical meristem.

To investigate the expression patterns of candidate *DWD* genes, we mined the publicly available transcript profiling data of soybean tissues at the Phytozome database (http://www.phytozome.net). As shown in Fig 4, all *DWD* genes displayed tissue-specific expression patterns. None of the gene was expressed ubiquitously in all the tissues under study suggesting that these genes have unique role in the tissue that they are expressed in. Thirty five *GmDWD* genes showed higher transcript accumulation in seeds. Similarly, 12 *GmDWD* genes in pods, 30 genes in flower, 27 genes in leaf, 10 genes in stem, 17 genes in shoot apical meristem, 25 genes in root, and 5 in nodule accumulated highest level of transcripts (Fig 4 and S5 Table). The maximal fold change for each of *DWD* genes was calculated using the ratio of the maximal and minimal FPKM in different tissues. It was found that the maximal fold change among different tissues ranged from 1.34 to 119.62 (the expression of *Glyma*.*15G138800* and *Glyma*.*12G155700* were undetectable in some tissues), and 125 *GmDWD* genes showed more than 2.0 maximal fold changes (S5 Table). Noticeably, 10 *GmDWD* genes were highly expressed in one or few tissues including *Glyma*.*06G046400*, *Glyma*.*06G185900*, *Glyma*.*06G242100*, *Glyma*.*07G058200*, *Glyma*.*12G155700*, *Glyma*.*13G184300*, *Glyma*.*13G329300*, *Glyma*.*15G138800*, *Glyma*.*16G027200*, and *Glyma*.*20G126400*, suggesting a potential function in the specific tissue (s). For example, *Glyma*.*15G138800* was mainly expressed in nodules, whereas its transcript accumulation was very low or undetectable in other tissues included in the study (Fig 4 and S5 Table), implying its potential function in nodule-related processes. Similarly, expression of *Glyma*.*06G046400* was distinct in flower among other tissues, suggesting that this gene might play an important role in the regulation of flower-related processes. Further observation indicated that not all the closely-related *DWD*s in phylogenetic tree were clustered together showed similar expression pattern. For example, 4 *GmDWD* genes (*Glyma*.*01G067900*, *Glyma*.*02G124400*, *Glyma*.*08G180700* and *Glyma*.*15G051700*) encoding putative mRNA export protein varied in their transcript accumulation profile (Fig 4).

### Gm08DWD interacts with GmMYB176

Previously we demonstrated that GmMYB176 regulates isoflavonoid biosynthesis by activating the *GmCHS8* gene expression [30]. To identify the proteins that interact with GmMYB176, an Y2H screen was performed where GmMYB176 was used as the bait and protein from embryos (50–60 days after pollination) as the prey proteins. The analysis identified a GmDWD protein (Gma08g145700), Gm08DWD, as an interacting protein of GmMYB176.

**Fig 5:**
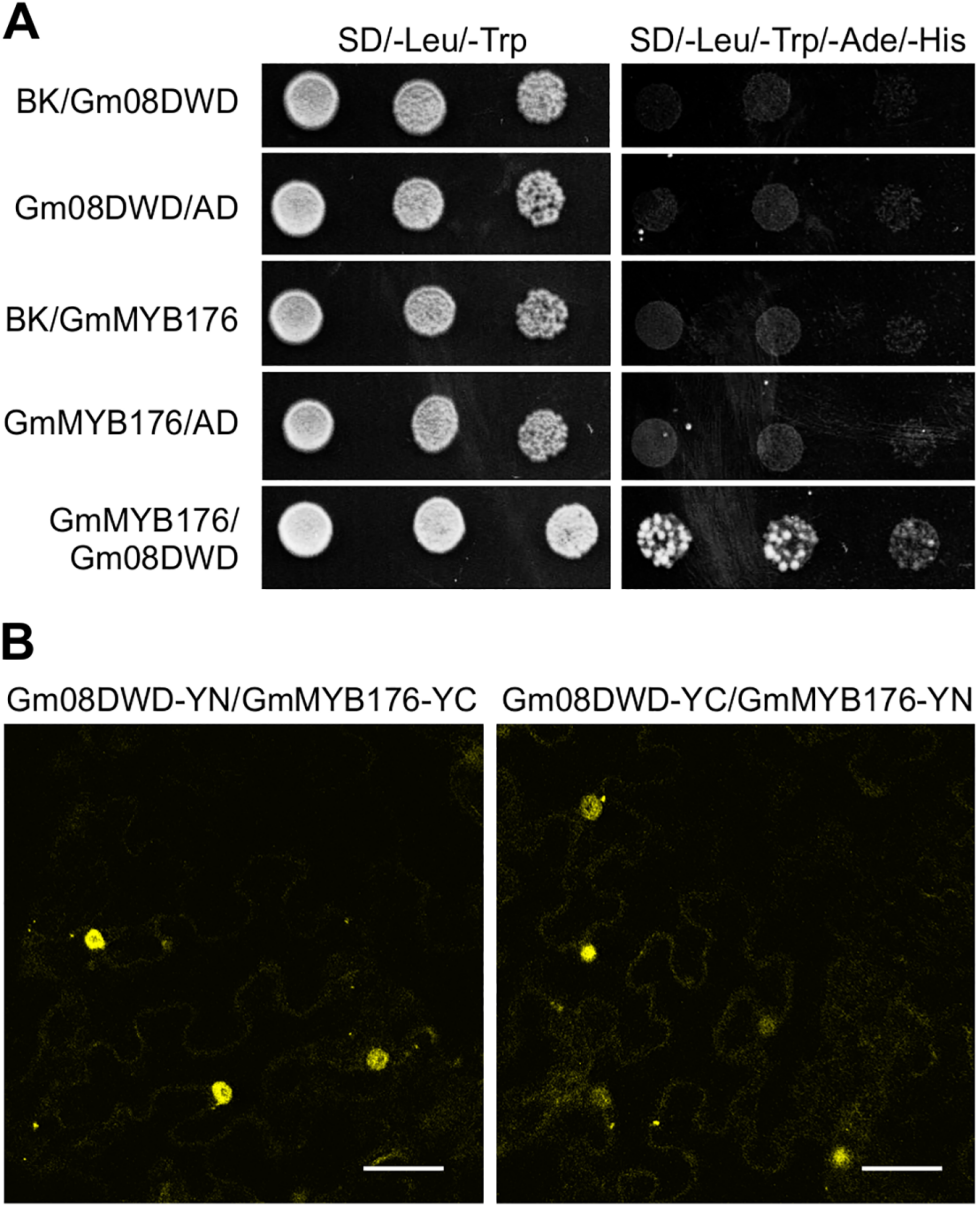
Interaction of Gm08DWD with GmMYB176. (A) A yeast two-hybrid assay showing interaction between Gm08DWD and GmMYB176 Yeast cells were co-transformed with combination of DNA-binding domain (BK, Bait) and activation domain (AD, Prey) fused constructs as indicated. A series of 5 μL of diluted yeast suspension culture co-transformed with bait and prey constructs was spotted onto synthetic defined (SD) selection plates. Growth on SD lacking adenine, histidine, leucine and tryptophan (–Ade/–His/–Leu/–Trp) requires activation of the reporter and indicates interaction. GmMYB176/BK, GmMYB176/AD, Gm08DWD/BK and Gm08DWD/AD are negative controls. (B) The BiFC assay showing interaction between Gm08DWD and GmMYB176 Tobacco leaves were co-transformed with GmMYB176 and Gm08DWD proteins fused to the N- or C-terminal half of yellow fluorescent protein followed by confocal microscopy.

To validate the result from the Y2H screening, a targeted Y2H assay was performed where GmMYB176 was used as the bait and Gm08DWD as the prey protein. As shown in Fig 5A, yeast colony growth was observed on the selective medium lacking Leu/Trp/Ade/His when the both the plasmids containing GmMYB176 as bait and Gm08DWD as prey were present, indicating the physical interaction between GmMYB176 and Gm08DWD proteins.

To confirm the interaction between GmMYB176 and Gm08DWD *in planta*, a BiFC analysis was carried out where split fluorescent protein segments were brought together to form a functional protein due to protein-protein interaction as described in detail previously [40]. Translational fusion of Gm08DWD or GmMYB176 was generated in the binary vector that contained either N-terminus half (YN) or C-terminus half (YN) of YFP. Tobacco leaves were co-infiltrated with *A*. *tumefaciens* containing pGm08DWD-YN and pGmMYB176-YC or pGmMYB176-YN and pGm08DWD-YC, and protein expression was monitored in leaf epidermal cells by confocal microscopy. The negative controls included the following combinations: (i) Gm08DWD-YN or -YC with the non-fusion half of YFP, (ii) GmMYB176-YN or -YC with the non-fusion half of YFP, and (iii) two non-fusion halves of YFP, YN and YC. As shown in Fig 5B, the interaction between Gm08DWD and GmMYB176 was confirmed *in planta* and the YFP signals were observed in the nucleus. The negative controls showed no signal (data not shown).

### Characterization of *Gm08DWD* gene

**Fig 6:**
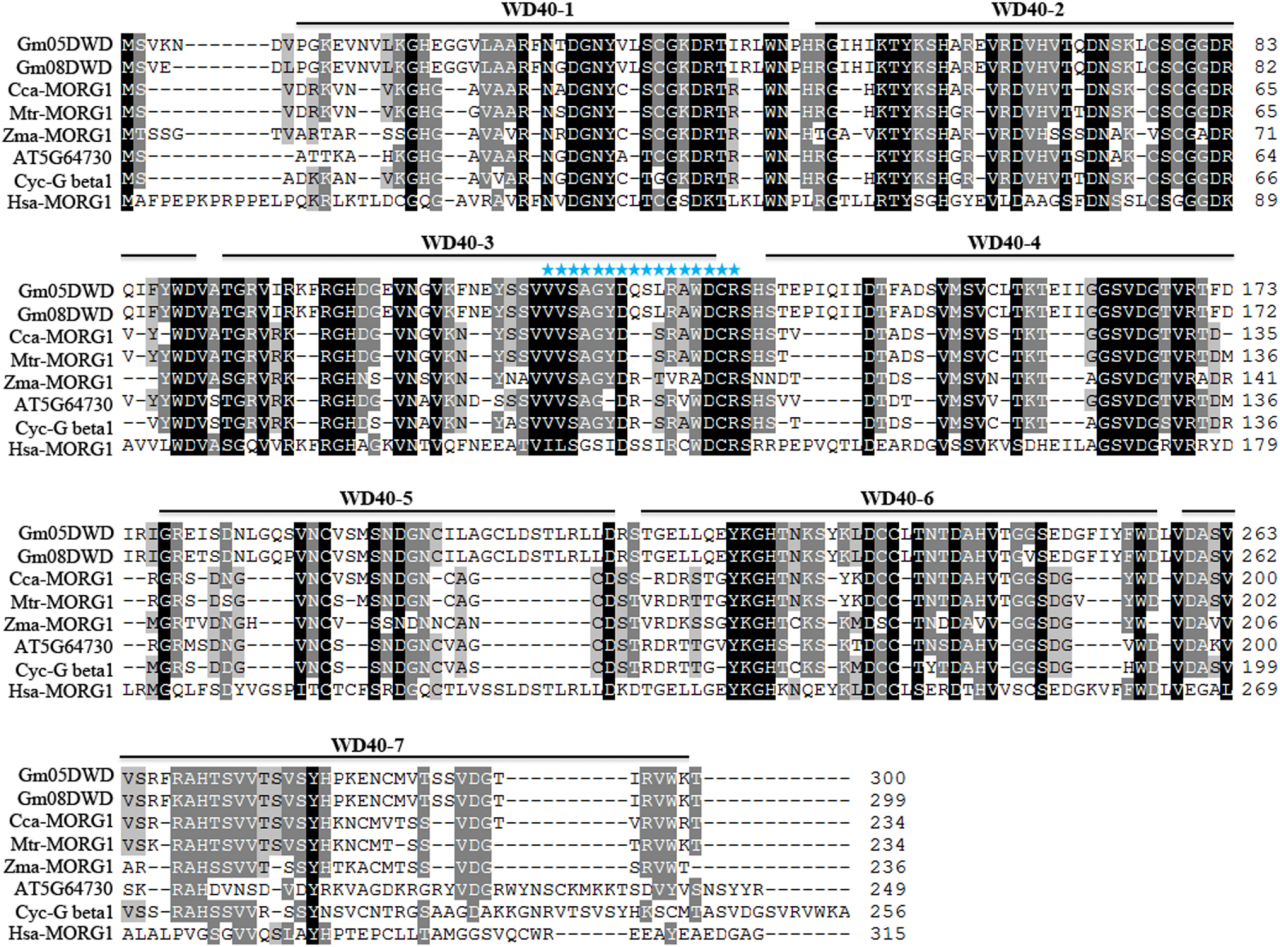
Multiple sequence alignment of deduced amino acid sequence of Gm08DWD with its homologs in different species. Amino acid sequences of Gm05DWD (Glyma05G187400), Gm08DWD (Glyma08G145700), Cca-MORG1 (*Cajanus cajan*, KYP67816), Mtr-MORG1 (*Medicago truncatula*, XP_013446856), Zma-MORG1 (*Zea mays*, NP_001141954), AT5G64730 (Arabidopsis WD40 repeat-like superfamily protein) Cyc-Gbeta1 G-protein beta 1 in *Cynara cardunculus*, KVI09821), Has-MORG1 (*Homo sapiens*, NP_001093207) were aligned by ClustalW, and imported in Genedoc for shading. Identical amino acid residues are shown in black. If the conserved percent was up to 80% and 60%, the amino acid residues were shaded with gray and light gray colors, respectively. The WD40 domains of candidate GmDWDs are indicated by lines and numbered. The DWD signature sequences of GmDWDs are marked with stars.

The full-length *Gm08DWD* cDNA sequence (966 nucleotides) was predicted to encode a protein of 321 amino acid residues with a calculated molecular mass of 35.8 kDa and a pI of 5.2. It consists of 8 exons and 7 introns and is located on chromosome 8 in soybean (Fig 3). The SMART analysis revealed 7 WD40 domains in Gm08DWD, with a single DWD motif within its third WD40 domain (Fig 6, Type A in Fig 1).

The phylogenetic analysis grouped Gm08DWD together with Gm05DWD (Glyma.05G187400) that resides on the chromosome 5 (Figs 2 and 3). Both of them map on the duplication block 570 with 93.9% sequence identity at nucleotide level, suggesting they are duplicated gene pair. Both Gm05DWD and Gm08DWD were annotated as Mitogen-activated protein kinase organizer 1 (MORG1), and share 97.7% sequence identity at amino acid level. MORG1 has been reported to act as modular scaffold involved in various processes in human and animal [47, 48]. To provide some functional clue of Gm08DWD and Gm05DWD, we obtained MORG1-like sequences from other species including human, and performed a multiple sequence alignment. The result indicated that they are highly conserved with 45.3–97.7% sequence identity (Fig 6).

**Fig 7:**
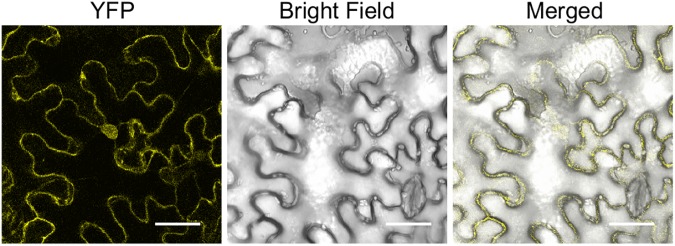
Subcellular localization of Gm08DWD. *A*. *tumefaciens* GV3101 carrying the plasmid with Gm08DWD-YFP fusion construct was infiltrated in *N*. *benthamiana* leaves and visualized by confocal microscopy. Scale bar is shown in μm.

To examine the subcellular localization of Gm08DWD, a translational fusion of Gm08DWD with YFP was created and transiently expressed in tobacco leaf epidermal cells. As shown in Fig 7, Gm08DWD was localized both in the nucleus and the cytoplasm. The intensities of YFP signals were similar in both the subcellular compartments.

**Fig 8:**
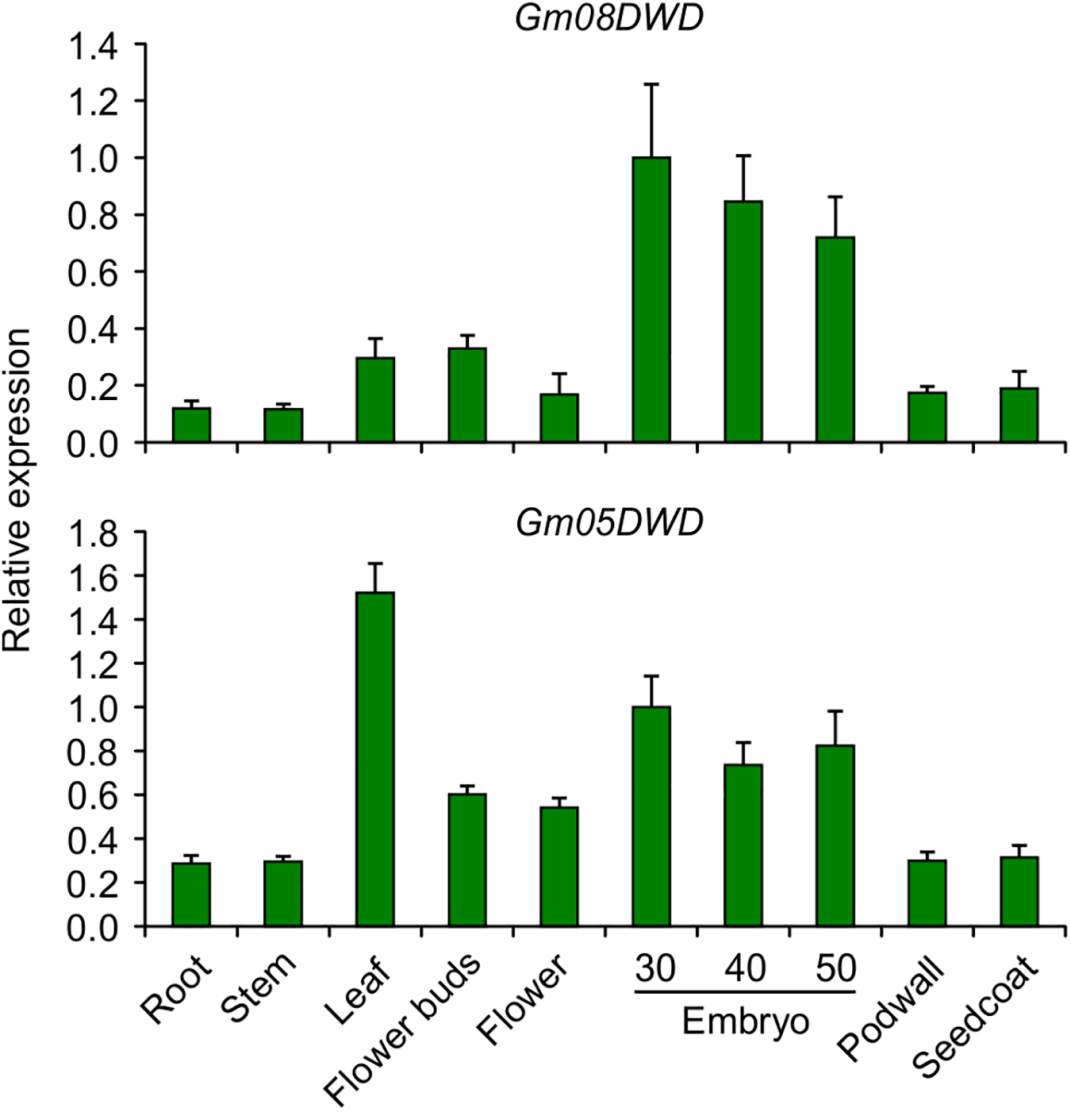
Expression analysis of *Gm05DWD* and *Gm08DWD* genes in soybean. Total RNA extracted from soybean root, stem, leaf, flower bud, flower, embryo (30, 40 and 50 days after pollination), seed coat and pod wall were used for quantitative RT-PCR analysis. Two biological replicates and three technical replicates for each biological replicate were carried out. The standard error of the mean is represented by an error bar. The data were normalized against *SUBI-3* gene.

**Fig 9:**
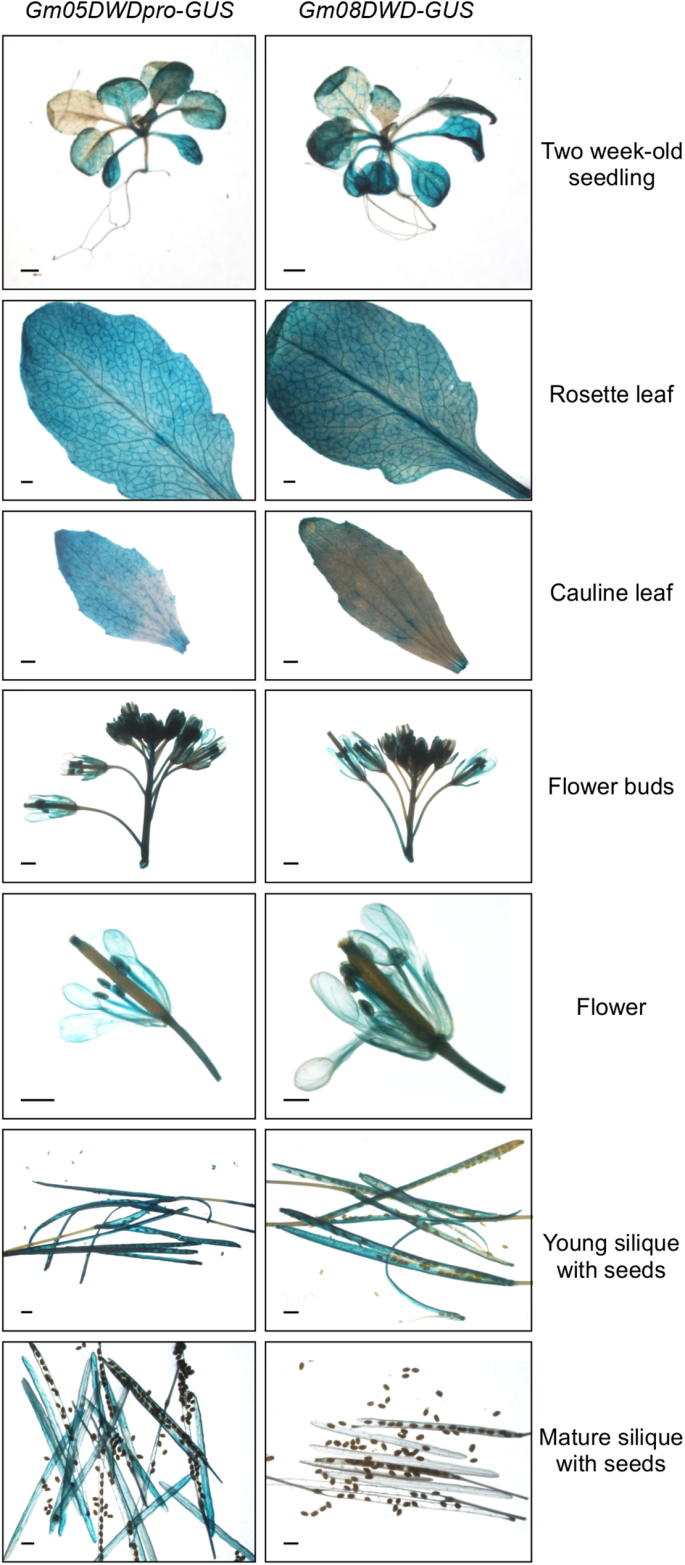
Histochemical analysis of *Gm05DWD*pro-GUS and *Gm08DWD*pro-GUS activity in *Arabidopsis*. Constructs containing either *Gm05DWD* or *Gm08DWD* promoter driven *GUS* gene were transformed into *Arabidopsis* and selected T2 transgenic plants were used for analysis in vegetative and reproductive tissues during various stages of development.

Despite of their high sequence similarity, *Gm05DWD* and *Gm08DWD* were expressed differentially in soybean tissues as indicated by the qPCR analysis using gene-specific primers. As shown in Fig 8, *Gm08DWD* expression was detected in root, stem, leaf, flower buds, flower, pod walls and seed coat tissues, however, the transcripts accumulated to higher levels in the developing embryos. In contrary, *Gm05DWD* accumulation was highest in the leaf tissue followed by developing embryos (Fig 8). To determine the spatial expression pattern of *Gm05DWD* and *Gm08DWD* genes in detail, we cloned the promoter regions of each of the genes covering 1.28 kb upstream of translational start site to drive *GUS* reporter gene, and transformed into wild-type *Arabidopsis* Col-0. Transgenic lines were selected for *Gm05DWDpro*:*GUS* and *Gm08DWDpro*:*GUS* and analysis of GUS expression was conducted in T_2_ generation by histochemical staining using multiple independent transgenic lines. The results indicated that strong GUS staining was detected in seedling, rosette leaf, stigma, filament, anther, flower stalk, pod wall for both transgenic plants (Fig 9), suggesting that *Gm05DWD* and *Gm08DWD* might perform similar functions in these tissues. Different GUS staining patterns were observed in seeds of two transgenic plants. As shown in Fig 9, GUS staining was clearly observed in young seeds of *Gm05DWD* transgenic plants, whereas relatively weak GUS staining was detected in mature seeds. In contrast, young seeds of *Gm08DWD* transgenic plants showed very weak GUS staining, while clear GUS staining was observed in mature seeds. These observations indicated that *Gm05DWD* and *Gm08DWD* might play different roles during seed development.

## Discussion

DWD proteins are encoded by a large multigene family in plants. In Arabidopsis, 85 DWD proteins were identified based on their conserved 16-amino acid residues [14]. Among them, 27 DWD proteins have been experimentally verified for their function as substrate receptors of CUL4-DDB1 [9, 16–18]. In this study, we identified 161 putative DWD protein encoding genes in soybean genome. It has been proposed that the DWD box within the WDR proteins binds to DDB1 proteins [4–6]. Although we did not experimentally define their interactions with DDB1, 62 GmDWDs were found to be orthologous to the DDB1 interactors in Arabidopsis such as COP1, SPAs, MSIs, DWAs (Fig 3, S1 and S2 Tables). Besides, 130 putative GmDWD proteins have their orthologs in Arabidopsis (S1 Table), which have identified as DWD protein by Lee et al [14]. These findings suggest that the DWD proteins identified in this study may have potential to bind to CUL4-DDB1 complex. However, additional protein lacking DWD motif can also bind to the CUL4-DDB1 complex [4, 10, 49], implying the underestimation of the number of soybean DDB1-binding proteins.

It has been proposed that DWD proteins can interact with DDB1 and serve as the substrate-recognition subunits of the CUL4-DDB1 ubiquitin E3 complex, and DWD motif is required for efficient DDB binding. Majority of putative DWD proteins in soybean (77.6%) contain single DWD motif (Fig 1), suggesting that one copy is sufficient to bind DDB1. However, 34 putative DWD proteins consisted of 2 DWD motifs, and 2 putative DWD proteins consisted of 3 DWD motifs within the sequence. This finding is consistent with the previous report that DWD proteins usually possess 1 and sometimes 2 but rarely 3 DWD motifs [5]. It has been suggested that the additional DWD motifs may enhance DDB1 binding or may interact with other proteins [14]. Besides WD40 domain, 29 DWD proteins contain several other known functional domains (Fig 1), which might be involved in interaction with substrate or other components. The presence of additional domains in the 29 soybean DWD proteins supports the hypothesis that the DWD motif binds DDB1 while other portions of the protein may bind substrates [14]. The remaining 132 putative GmDWDs do not possess any additional domains. It is possible that these DWDs bind their substrates with some unidentified motif. The features of DWD proteins such as the large number, diverse domains and multiple DWD motifs suggest functional diversity in some aspects, and is consistent with the proposal that CUL4-mediated ubiquitin E3 complex regulates diverse processes such as RNA processing, protein assembly and degradation, signal transduction, epigenetic regulation, cell cycle progression, cytoskeletal dynamics (S1 Fig). Although 26.1% GmDWDs have not been annotated or characterized yet, their Arabidopsis orthologs identified in this study provides clues for their possible functions. Additionally, *DWD* genes displayed tissue-specific expression patterns. Ten putative *GmDWD* genes were highly expressed in one or few tissues only while others were expressed in multiple tissues (Fig 4). It has been proposed that gene expression provides functional specificity in certain tissues [50]. Diverse expression patterns in soybean tissues indicated functional diversification among *GmDWD* genes.

Soybean *DWD* genes were unevenly distributed on the 20 chromosome. Evidently, some chromosomes exhibited dense distribution of *DWD* genes, whereas others contained sparse distribution of them (Fig 3). Similar *DWD* gene distribution on chromosomes was reported in foxtail millet [12] and tomato [15]. The uneven distribution of the *DWD* genes suggested diverse contributions of soybean chromosomes to the formation and expansion of *DWD* gene family. It has been estimated that soybean genome has undergone two whole genome duplication events approximately 56.5 and 19.2 million years ago [51]. This has resulted into duplication of at least 75% of gene in soybean genome [52]. Here, we identified 48 duplicated gene pairs within the 161 *GmDWD*s. Among them, 3 pairs of *GmDWD* genes were likely derived from tandem amplification and 45 pairs of *GmDWD* genes from segmental duplication (S4 Table). Each pair of duplicated genes formed a discrete clade in the phylogenetic tree with 75.5–99% sequence identity (Fig 2), indicating a closely-related evolutionary relationship. Evidently, the origins of these putative *GmDWD* genes are in agreement with soybean evolutionary history. In addition, their low Ka/Ks values implied that these duplicated genes might have undergone a purifying selection with limited functional divergence after duplication. Taken together, the gene duplication and sequence identity together with low Ka/Ks values suggest that each pair of *GmDWD* duplications or amplifications possibly share similar function with each other. Intriguingly, 14 pairs of duplicated genes shared similar expression pattern in different tissues (Fig 4 and S4 Table). Since functional role of a gene can be reflected by their temporal and spatial expression [50], we speculate that the 14 pairs of duplicated genes may display functional redundancy in soybean.

It has been demonstrated that plant DWD proteins play important roles in diverse processes, including the regulation of photomorphogenesis and flowering time [19, 24], signal transduction [22, 23], chromatin modification [20], stress response [9, 53], as well as gametophyte [21], embryo and endosperm development [17]. In this study, we investigated the interaction of Gm08DWD with an isoflavonoid regulator GmMYB176 and speculated the possible consequence in isoflavonoid biosynthesis. Previously we demonstrated that GmMYB176 regulates *CHS8* gene expression and affects isoflavonoid biosynthesis in soybean seeds [30]. We also revealed that 14-3-3 proteins regulate the intracellular localization of GmMYB176 thereby affecting isoflavonoid biosynthesis in soybean [40]. To further dissect the mechanisms of GmMYB176-mediated regulation of isoflavonoid biosynthesis of soybean seeds, an Y2H assay was performed using GmMYB176 as the bait and proteins from developing embryo as the prey. Here we demonstrate the interaction of GmMYB176 with Gm08DWD (Fig 5). Furthermore, *Gm08DWD* showed a high expression level in soybean embryo and Arabidopsis mature seeds (Figs 8 and 9), which is consistent with the previous report that the expression of *GmMYB176* is relatively high in soybean embryo. The similar expression patterns may imply an association between these two genes during seed development and maturation. Based on these evidences, we speculated that Gm08DWD might be involved in the regulation of isoflavonoid biosynthesis in soybean seeds through its interaction with GmMYB176. Since Gm08DWD is putatively a DWD protein and has 81.2% identity with its Arabidopsis homologue which is predicted as a component of CUL4-DDB1complex and G protein complex in Arabidopsis, we speculate its similar function in soybean. In Mammalian cells, MORG1 was found to interact with PHD3, thereby regulating the protein levels of HIF-1α via proteasome degradation pathway [47]. MORG1 has not been functionally characterized in plants, however, sequences similar to MORG1 has been identified in many plant species including Gm08DWD in soybean (Fig 6). The fact that GmMYB176 and Gm08DWD interact with each other and the site of interaction is nucleus (Fig 5), is consistent with the functional localization of CUL4-E3 ligase complex [53]. Thus, we speculate that CUL4-DDB1 complex or other protein complex might specifically recognize the GmMYB176 via its specific substrate, Gm08DWD, thereby regulating the degradation of GmMYB176 through the ubiquitination pathway. Further experimental evidences are required to support this speculation.

## Supporting information

S1 Fig Functional categories of DWD proteins in soybean. (TIF)

S1 Table List of primers used in the study. (DOCX)

S2 Table List of candidate GmDWD proteins and their homologs in Arabidopsis. (DOCX)

S3 Table List of soybean DWD proteins which homologs are not identified. (DOCX)

S4 Table Segmental duplication events during evluotion of *DWD*-containing genes in soybean. (XLSX)

S5 Table Trancript profile data of 161 *DWD* genes in soybean. (XLSX)
