# Supplementary figures and images for: Genome-wide analysis of DWD proteins in soybean (Glycine max): Significance of Gm08DWD and GmMYB176 interaction in isoflavonoid biosynthesis
Source: PLoS One. 2017 Jun 6;12(6):e0178947. doi: 10.1371/journal.pone.0178947 (PMC5460815; doi:10.1371/journal.pone.0178947)

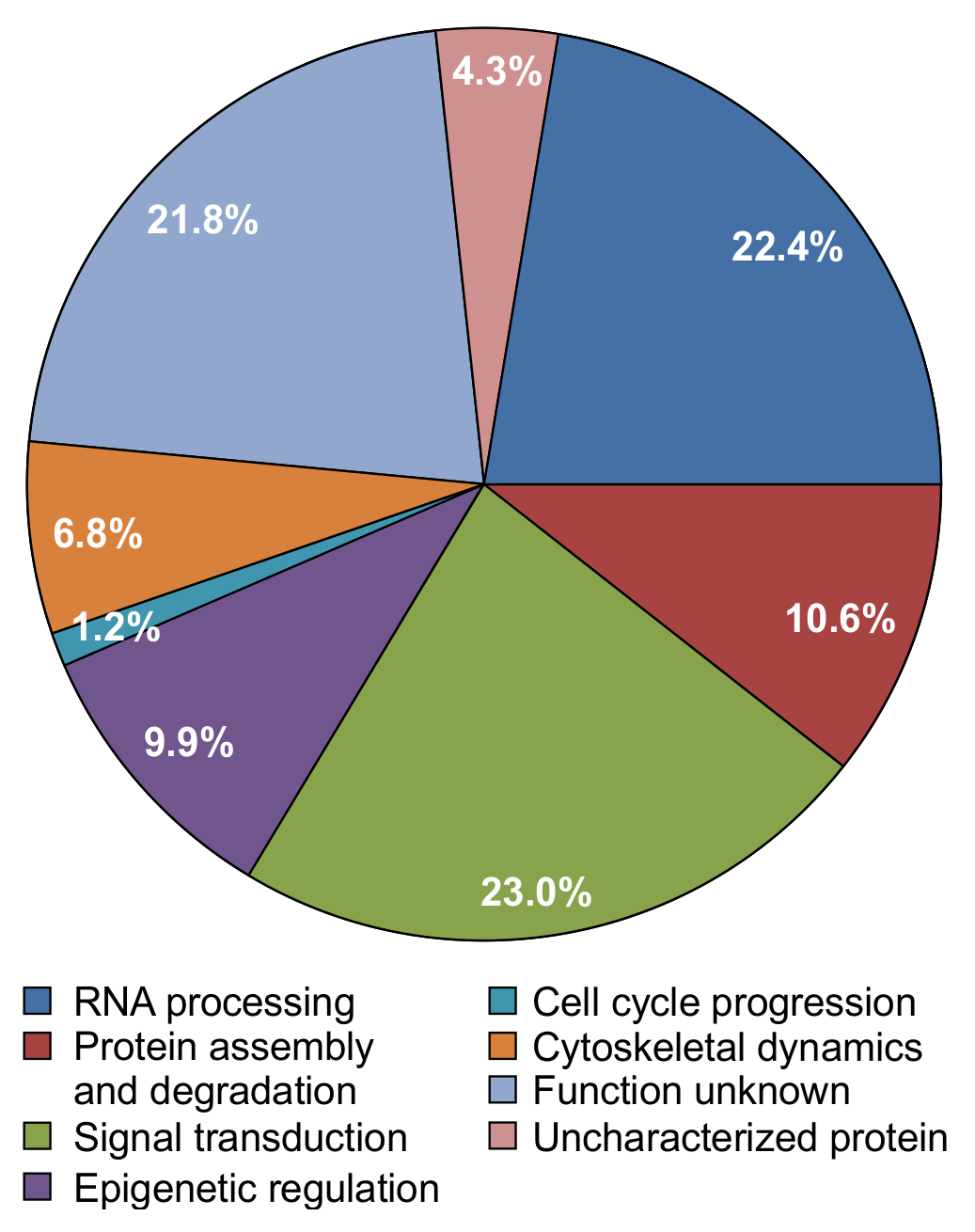

Supplement: S1 Fig — (TIF) [file pone.0178947.s001.tif]
