# Supplementary material for: Genome-wide analysis of DWD proteins in soybean (Glycine max): Significance of Gm08DWD and GmMYB176 interaction in isoflavonoid biosynthesis
Source: PLoS One. 2017 Jun 6;12(6):e0178947. doi: 10.1371/journal.pone.0178947 (PMC5460815; doi:10.1371/journal.pone.0178947)
Supplement: S1 Table — (DOCX) [file pone.0178947.s002.docx]

| **Gene name** | **Primer name** | **Sequence (5'-3')** | **Purpose** |
| --- | --- | --- | --- |
| *Gm08DWD* | Gm08DWD-F | ATTGCGTTTGGAGTTCACGGACA |  |
|  | Gm08DWD-R | ACCATCTTCCACATGAAAAGCACATA |  |
| *Gm05DWD* | Gm05DWD-F | AGCTTATATGGTGTGTCCACGCAGC  qPCR analysis |  |
|  | Gm05DWD-R | GTAACATCGTGTCTGTGGACTCCATAT |  |
| *SUBI3* | SUBI3-F | GTGTAATGTTGGATGTGTTCCC |  |
|  | SUBI3-R | ACACAATTGAGTTCAACACAAACCG |  |
| *Gm08DWD* | Gm08DWD-gF | ggggacaagtttgtacaaaaaagcaggcttcATGAGCGTGGAAGATCTTCC | Gene cloning |
|  | Gm08DWD-gR | ggggaccactttgtacaagaaagctgggtcTGTCTTCCATACTCTAATGGTGCC |  |
| *Gm05DWD* | Gm05DWD-gF | ggggacaagtttgtacaaaaaagcaggctcATGAGCGTGAAAAATGATGTTCC |  |
|  | Gm05DWD-gR | ggggaccactttgtacaagaaagctgggtcTGTCTTCCATACTCTAATGGTGCC |  |
| *Gm08DWD* | Gm08DWD-pF | ggggacaagtttgtacaaaaaagcaggctcTTACACTTGTTCTTATCTCG | Promoter cloning |
|  | Gm08DWD-pR | ggggaccactttgtacaagaaagctgggtcTCTTCTTCTTCTTCTTCTTCTTC |  |
| *Gm05DWD* | Gm05DWD-pF | ggggacaagtttgtacaaaaaagcaggctcTGATATCGTATATTGTTGGACGC |  |
|  | Gm05DWD-pR | ggggaccactttgtacaagaaagctgggtcTCTTCTACTTCACTAGGCTCT |  |

**Table S1** List of primers used in the study

Lowercase nucleotides in the primer sequences indicate *att* sites for Gateway cloning.
