# Supplementary material for: Genome-wide analysis of DWD proteins in soybean (Glycine max): Significance of Gm08DWD and GmMYB176 interaction in isoflavonoid biosynthesis
Source: PLoS One. 2017 Jun 6;12(6):e0178947. doi: 10.1371/journal.pone.0178947 (PMC5460815; doi:10.1371/journal.pone.0178947)
Supplement: S2 Table — (DOCX) [file pone.0178947.s003.docx]

**Table S2** List of candidate GmDWD proteins and their homologs in Arabidopsis

| Gene ID | DWD Motif^a^ | Gene Annotation | Homolog in Arabidopsis^b^ | Gene | Type^c^ |
| --- | --- | --- | --- | --- | --- |
| Glyma.01G006600 | (1087) FVSASDDSTVKIWDSR | Phosphoinositide-3-kinase | AT4G29380 (70/99) | *ATVPS15* | I |
| Glyma.01G029600 | (206) FLTGSLDKSARLWDTR | Translation initiation factor 3 | AT2G46290 (81/100) | *WD40 Family* | A |
| Glyma.01G067900 | (127) LASGSWDKTLKYWDTR | mRNA export protein | AT1G80670 (83/100) | *RAE1* | A |
| Glyma.01G086000 | (379) LLSASYDGKVMLWDLR  (330) IAAGGSDPVIRIWDPR | Microtubule binding protein YTM1 | AT5G15550 (71/98) | *ATPEP2* | M |
| Glyma.01G224200 | (811) LAFGSADYSTYCYDLR | U5 snRNP-specific protein-like factor | AT4G11110 (60/89) | *SPA2* | A |
| Glyma.02G012900 | (344) LLSSSDDGTCRIWDAR | WD40 repeat protein | AT2G47410 (69/79) | *WD40 Family* | A |
| Glyma.02G080600 | (424) LLSGSKDSTLKVWDIR | Notchless-like WD40 repeat protein | AT5G52820 (79/96) | *ATNLE* | A |
| Glyma.02G097500 | (328) FATGNQDKTCRVWDVR | WD40 repeat protein | AT5G56190 (62/99) | *WD40 Family* | A |
| Glyma.02G124400 | (127) LATGSWDKTLKYWDTR | mRNA export protein | AT1G80670 (84/100) | *RAE1* | A |
| Glyma.02G146800 | (130) IVSGSFDETIKVWDVK  (172) IISASHDGSCKIWDTR | WD40 repeat protein | AT4G02730 (69/99) | *ATWDR5B* | M |
| Glyma.02G151500 | (186) FVTGSYDHVVRLWDAR | U3 small nucleolar RNA-associated protein 15 | AT2G47990 (58/100) | *EDA19* | A |
| Glyma.02G226300 | (326) FLTCGNDHFARIWDLR | DNA damage-binding protein 2 | AT5G58760 (74/89) | *DDB2* | G |
| Glyma.02G251700 | (219) LATSASDRSIILYDLR  (261) FTAANEDGNCYSYDAR | WD repeat and SOF domain-containing protein 1 | AT4G28450 (77/100) | *DWD motif* | M |
| Glyma.02G267800 | (557) LASASTDSTLRLWDVK  (515) IAVGSADHHIHYYDLR | E3 ubiquitin-protein ligase RFWD2 | AT2G3295 (77/100) | *ATCOP1* | S |
| Glyma.03G243800 | (233) LVSGGTEKVVRVWDAR | WD40 repeat protein | AT3G05090 (71/100) | *LRS1* | A |
| Glyma.03G244500 | (233) LVSGGTEKVVRVWDTR | WD40 repeat protein | AT3G05090 (71/99) | *LRS1* | A |
| Glyma.04G018500 | (183) SVAATCESYLQFWDVR  (224) LVTAEHESGIHIWDLR | WD repeat protein TSSC1 | AT1G20540 (76/99) | *WD40 Family* | M |
| Glyma.04G045700 | (218) LVTGGCDGAIRFWDIR | DNA excision repair protein ERCC-8 | AT1G27840 (67/98) | *ATCSA-1* | A |
| Glyma.04G093100 | (232) LLVGGSDAFARLYDRR  (654) VASGSDDGRWYIWEKR | WD and tetratricopeptide repeats protein 1 | AT5G10940 (66/100) | *ASG2* | M |
| Glyma.04G104200 | (335) LATGNQDKTCRLWDIR | WD40-like repeat containing protein | AT1G36070 (69/92) | *WD40 Family* | A |
| Glyma.04G112700 | (333) FATGNQDKTCQIWDLR | WD40 repeat protein | AT5G56190 (72/99) | *WD40 Family* | A |
| Glyma.05G027800 | (368) LLSASYDGKVMLWDLR | Microtubule binding protein YTM1 | AT5G15550 (70/99) | *ATPEP2* | A |
| Glyma.05G004600 | (147) IVSGSRDGSFRIWDLR  (217) ATAGAVDSVLKFWDTR | Histone H3 (Lys4) methyltransferase complex | AT3G27640 (52/99) | *WD40 Family* | M |
| Glyma.05G100500 | (391) LLTGSCDQTAIVWDVK | WD40 repeat protein | AT5G67320 (69/100) | *HOS15* | E |
| Glyma.05G131200 | (243) FGSVGDDQYLLIWDLR | CAF1/NURF55/MSI1 | AT5G58230 (89/73) | *ATMSI1* | K |
| Glyma.05G147800 | (289) FASCSVDGNIAIWDTR | Ribosome Assembly protein | AT2G19540 (74/94) | *HTD1* | A |
| Glyma.05G170500 | (364) VITASSDCTIKVWDVK | SMU1 | AT1G73720 (87/99) | *SMU1* | L |
| Glyma.05G187400 | (118) VVSAGYDQSLRAWDCR | Mitogen-activated protein kinase organizer 1 | AT5G64730 (82/98) | *WD40 Family* | A |
| Glyma.06G013000 | (220) FVSGSCDSTARLWDTR | G protein | AT4G34460 (80/100) | *ATAGB1* | A |
| Glyma.06G018800 | (183) SVAATCESYLQFWDVR  (224) LVTAEHESGIHIWDLR | TSSC1 | AT1G20540 (77/79) | *WD40 Family* | M |
| Glyma.06G026400 | (235) VLAGYEDGSMLWWDVR | G-protein beta subunit-like protein GNB1L | AT4G29860 (63/99) | *ALT2,* | A |
| Glyma.06G046400 | (214) LVTGGCDGAIRFWDIR | DNA excision repairprotein ERCC-8 | AT1G27840 (66/97) | *ATCSA-1* | A |
| Glyma.06G094900 | (185) LLVGGSDAFARLYDRR | WD and tetratricopeptide repeats protein 1 | AT5G10940 (69/100) | *ASG2* | A |
| Glyma.06G105200 | (303) LATGNQDKTCRLWDIR | WD40-like repeat containing protein | AT1G78070 (67/100) | *WD40 Family* | A |
| Glyma.06G242100 | (540) IASSNFEGVVQLWDVTR | G-protein beta subunit | AT3G15354 (61/100) | *SPA3* | J |
| Glyma.07G058200 | (913) VVSASTDNSLKLWDLK  (871) LFFGSADYKVYGYDLR | eIF-2alpha kinase PEK/EIF2AK3 | AT2G46340 (48/91) | *SPA1* | P |
| Glyma.07G068400 | (314) IASCGLDSYLRLWDTK | Uncharacterized conserved protein | AT1G29320 (62/79) | *WD40 Family* | A |
| Glyma.07G125200 | (1096) FVSASDDSTVKIWDSR | Phosphoinositide-3-kinase, regulatory subunit 4 | AT4G29380 (70/100) | *ATVPS15* | I |
| Glyma.07G192100 | (85) FASGSSDTNLNIWDIR | Microtubule severing protein katanin p80 | AT1G61210 (56/87) | *DWA3* | A |
| Glyma.07G270300 | (351) FATGNQDKTCRVWDVR | WD40 repeat protein | AT3G13340 (73/95) | *WD40 Family* | A |
| Glyma.08G021200 | (799) LAFGSADYSAYCYDLR | eIF-2alpha kinase PEK/EIF2AK3 | AT4G11110 (52/100) | *SPA2* | A |
| Glyma.08G040500 | (956) VLTASHDGTVKMWDVR  (997) LAAAGRDVVANIWDIR | WD40 repeat protein | AT1G49040 (74/100) | *SCD1* | R |
| Glyma.08G085900 | (243) FGSVGDDQYLLIWDLR | CAF1/NURF55/MSI1 | AT5G58230 (90/99) | *ATMSI1* | A |
| Glyma.08G104700 | (274) FASCSVD GNIAIWDTR | Ribosome Assembly protein | AT2G19540 (68/93) | *HTD1* | A |
| Glyma.08G128700 | (364) VITASSDCTIKVWDVK | SMU1 | AT1G73720 (87/99) | *SMU1* | L |
| Glyma.08G145700 | (117) VVSAGYDQSLRAWDCR | Mitogen-activated protein kinase organizer 1 | AT5G64730 (82/99) | *WD40 Family* | A |
| Glyma.08G180700 | (127) LVTGSWDKTLKYWDTR | mRNA export protein | AT1G80670 (81/98) | *RAE1* | A |
| Glyma.08G230900 | (352) LLTGSYDEYLRVWDLR  (306) VYTGSDDCKFSCWDLR | Uncharacterized conserved protein | AT5G63010 (58/60) | *WD40 Family* | N |
| Glyma.08G336200 | (81) IATASGDKTVRLWDAR | THO complex subunit 3 | AT5G56130 (88/100) | *ATTEX1* | A |
| Glyma.09G017700 | (334) FATGNQDKTCRVWDVR | WD40 repeat protein | AT3G13340 (75/95) | *WD40 Family* | A |
| Glyma.09G023100 | (218) LATGGLDRHIHIWDTR | U3 snoRNP-associated protein | AT4G21130 (54/98) | *EMB2271* | A |
| Glyma.09G037300 | (347) LLSS SDDGTCRIWDAR | Protein binding | AT5G49430 (50/99) | *WD40 Family* | B |
| Glyma.09G043800 | (308) VVTGSHDTTIKMWDLR  (266) LLTGGRDSVCRVWDI R | Pleiotropic regulator 1 | AT4G15900 (79/99) | *PRL1* | M |
| Glyma.09G063100 | (309) FCSVGDDSCLILWDAR | CAF1/NURF55/MSI1 | AT2G19520 (83/96) | *ATMSI4* | A |
| Glyma.09G077900 | (227) LLTGSYDEFLRVWDLR | Uncharacterized conserved protein | AT5G63010 (60/99) | *WD40 Family* | A |
| Glyma.09G084600 | (450) LLSASLDGTIRAWDLLR | Periodic tryptophan protein 2 | AT1G15440 (72/99) | *ATPWP2* | A |
| Glyma.09G086200 | (269) VASASSDGIIRV WDVR  (217) LYTGGEDRNITAWDIK | WD40 repeat protein | AT1G65030 (62/93) | *DWD motif* | M |
| Glyma.09G234100 | (206) FLTGSLDKSARLWDTR | Translation initiation factor eIF-3 subunit 2 | AT2G46290 (80/100) | *WD40 Family* | A |
| Glyma.09G278500 | (1214) FTAGFIDGSVRLYDVR | Regulatory associated protein of mTOR | AT3G08850 (75/100) | *ATRAPTOR1B* | A |
| Glyma.10G023000 | (330) FISNGKDQTIKLWDIR  (284) IYSGSDDSFCKVWDRR | WD40 repeat protein 23 | AT4G03020 (73/99) | *WD40 Family* | M |
| Glyma.10G027100 | (130) IVSGSFDETIK VWDVK | WD40 repeat protein | AT4G02730 (70/94) | *ATWDR5B* | A |
| Glyma.10G133700 | (371) LGTGTTESLVKIWDVK | pre-mRNA-processing factor 19 | AT2G33340 (75/99) | *MAC3B* | D |
| Glyma.10G200300 | (295) MATAGKDKKIKLWDLR | WD40 repeat protein | AT3G10530 (69/98) | *WD40 Family* | C |
| Glyma.10G259600 | (603) FATSSFDHDVKMWDLR | WD40 REPEAT FAMILY | AT4G34280 (53/100) | *DHU1* | A |
| Glyma.11G018700 | (815) LAFGSADYSTYCYDLR | eIF-2alpha kinase PEK/EIF2AK3 | AT4G11110 (51/100) | *SPA2* | A |
| Glyma.11G091500 | (227) FGSGGDDCKLIIWDLR | CAF1/NURF55/MSI1 | AT2G16780 (67/98) | *MSI02* | A |
| Glyma.11G118500 | (220) FVSGSCDATARLWDTR | G protein beta 1 | AT4G34460 (81/99) | *ATAGB1* | A |
| Glyma.12G002900 | (206) FLTGSLDKSARLWDTR | Translation initiation factor eIF-3 subunit 2 | At2g46290 (80/100) | *WD40 Family* | A |
| Glyma.12G033100 | (225) FGSSGDDCKLIIWDLR | CAF1/NURF55/MSI1 | AT2G16780 (67/98) | *MSI02* | A |
| Glyma.12G043900 | (220) FVSGSCDATARLWDTR | G protein | AT4G34460 (81/99) | *ATAGB1* | A |
| Glyma.12G155700 | (589) LAFGSADHRIYYYDLR | WD40 repeat protein | AT3G15354 (62/99) | *SPA3* | J |
| Glyma.12G224600 | (637) LAFGSADHRIYYYDLR | E3 ubiquitin-protein ligase RFWD2 | AT3G15354 (64/99) | *SPA3* | J |
| Glyma.13G056900 | (376) LLSASYDGKVMLWDLR  (330) IAAGGSDPVIRIWDPR | Microtubule binding protein YTM1 | AT5G15550 (71/98) | *ATPEP2* | M |
| Glyma.13G106300 | (219) IITGSEDGTTRIWDCK | THO complex subunit 6 | AT2G19430 (65/98) | *DWA1* | A |
| Glyma.13G184300 | (115) FASGSLDTNLNIWDIR | Microtubule severing protein katanin p80 | AT1G61210 (61/86) | *DWA3* | A |
| Glyma.13G198200 | (340) VLTASKDQIIK LYDIR  (298) LVSGGKDNLVKLWDAK | Polyadenylation factor I complex, subunit PFS2 | AT5G13480 (66/92) | *FY* | M |
| Glyma.13G213100 | (313) LISNGKDQTTKLWDIR  (267) IYSGSDDSFIKVWDRR | WD40 repeat protein | AT4G03020 (71/99) | *WD40 Family* | M |
| Glyma.13G229400 | (209) IATASVDKSVKVWDVR  (166) FASASGDCTLRVWDVR | Peroxisomal targeting signal type 2 receptor | AT1G29260 (82/99) | *ATPEX7* | M |
| Glyma.13G244300 | (115) FASGSMDTNLKIWDIR | Microtubule severing protein katanin p80 | AT1G61210 (63/93) | *DWA3* | A |
| Glyma.13G255400 | (394) LLSGGGDGQVYHWDLR | U3 small nucleolar RNA-associated protein 18 | AT5G14050 (64/100) | *WD40 Family* | A |
| Glyma.13G329300 | (220) FAIGGSDEYARVYDMR | WD40 repeat protein | AT3G45620 (63/96) | *DWD motif* | A |
| Glyma.13G350500 | (258) FCSVGDDSRLILWDAR | CAF1/NURF55/MSI1 | AT2G19520 (77/99) | *ATMSI4* | A |
| Glyma.14G049700 | (563) LASASTDSTLRLWDVK  (521) IAVGSADHHIHYYDLR | E3 ubiquitin-protein ligase RFWD2 | AT2G32950 (78/100) | *ATCOP1* | O |
| Glyma.14G065100 | (220) LATSASDRSIILYDLR  (262) FTAANEDGNCYSYDAR | Sof1-like rRNA processing protein | AT4G28450 (75/100) | *DWD motif* | M |
| Glyma.14G103000 | (136) FISGS LDRTVLLWDQR | Histone H3 (Lys4) methyltransferase complex | AT5G66240 (86/87) | *ULCS1* | A |
| Glyma.14G193200 | (332) FLTCGNDHFARIWDLR | DNA damage-binding protein 2 | AT5G58760 (74/90) | *DDB2* | G |
| Glyma.15G024000 | (154) FCSVGDDSRLILWDAR | CAF1/NURF55/MSI1 | AT2G19520 (59/99) | *ATMSI4* | A |
| Glyma.15G044600 | (201) FAIGGSDEYA RVYDIR | WD40 repeat protein | AT3G45620 (63/99) | *DWD motif* | A |
| Glyma.15G051700 | (127) LVTGSWDKTMKYWDTR | mRNA export factor | AT1G80670 (81/98) | *RAE1* | A |
| Glyma.15G059400 | (416) LLSSGGDGQVYHWDLR | U3 small nucleolar RNA-associated protein 18 | AT5G14050 (63/100) | *WD40 Family* | A |
| Glyma.15G069400 | (115) FASGSMDTNLKIWDIR | Microtubule severing protein katanin p80 | AT1G61210 (63/99) | *DWA3* | A |
| Glyma.15G083100 | (209) IATASVDKSVKVWDVR  (166) FASASGDCTLRVWDVR | Peroxisomal targeting signal type 2 receptor | AT1G29260 (78/99) | *ATPEX7* | M |
| Glyma.15G099700 | (313) LISNGKDQTTKLWDIR  (267) IFSGSDDSFIKVWD RR | WD40 repeat protein | AT4G03020 (74/93) | *WD40 Family* | M |
| Glyma.15G123600 | (334) FATGNQDKTCRVWDVR | WD40 repeat protein | AT3G13340 (75/99) | *WD40 Family* | A |
| Glyma.15G138800 | (304) FATGNRDRTCRIWDAR | G-protein beta subunit-like protein | AT3G13340 (49/99) | *WD40 Family* | A |
| Glyma.15G142600 | (302) LLSSSDDGTCRIWDAR | WD40 repeat protein | AT2G47410 (73/74) | *WD40 Family* | B |
| Glyma.15G149300 | (307) VVTGSHDTTIKMWDLR  (265) LLTGGRDSVCRVWDIR | Pleiotropic regulator 1 | AT4G15900 (79/99) | *PRL1* | M |
| Glyma.15G169800 | (304) FCSVGDDSCLILWDAR | CAF1/NURF55/MSI1 | AT2G19520 (83/96) | *ATMSI4* | A |
| Glyma.15G193000 | (444) LLSASLDGTIRAWDLLR | WD40 repeat protein | AT1G15440 (70/96) | *ATPWP2* | A |
| Glyma.15G195000 | (269) VASASSDGIIRV WDVR (217) LYTGGEDRNITAWDIK | WD40 repeat protein | AT1G65030 (61/94) | *DWD motif* | M |
| Glyma.15G218700 | (227) LLTGSYDEFLRVWDLR (181) VYTGSDDCKFSCWDLR | Uncharacterized conserved protein | AT5G63010 (61/99) | *WD40 Family* | M |
| Glyma.15G236500 | (341) VLTASKDQIIKLYDIR  (299) LVSGGKDNLVKLWDAK | Polyadenylation factor I complex, subunit PFS2 | AT5G13480 (76/64) | *FY* | M |
| Glyma.16G027200 | (801) LFFGSADYKVYGYDLR | Peroxisomal targeting signal type 2 receptor | AT2G46340 (49/87) | *SPA1* | J |
| Glyma.16G034400 | (317) IASCGLDSYLRLWDTK | Uncharacterized conserved protein | AT1G29320 (63/79) | *WD40 Family* | A |
| Glyma.16G037700 | (197) IFTGGIDNDVKIWDLR  (156) VVSGSDDGTAKLWDMR  (239) LLTNGMDCKLCIWDMR | U5 snRNP-specific protein-like factor | AT2G43770 (86/100) | *WD40 Family* | T |
| Glyma.16G071900 | (132) FMSGSLDHSVRIWDLR | Histone H3 (Lys4) methyltransferase complex | AT5G14530 (82/94) | *WD40 Family* | A |
| Glyma.16G165100 | (424) LLSGSKDSTLKVWDIR | Notchless-like WD40 repeat-containing protein | AT5G52820 (78/96) |  | A |
| Glyma.17G003600 | (334) FATGNQDKTCRVWDVR | WD40 repeat protein | AT3G13340 (77/93) | *WD40 Family* | A |
| Glyma.17G053100 | (219) IITGSEDGTTRIWDCK | THO complex subunit 6 | AT2G19430 (65/98) | *DWA1* | A |
| Glyma.17G103900 | (307) VATSSTDGTACTWDLR | WD40 repeat protein | AT1G80710 (45/98) | *DRS1* | A |
| Glyma.17G165900 | (381) LLTGSCDQTAIVWDVK | WD40 repeat protein | AT5G67320 (70/100) | *HOS15* | E |
| Glyma.17G222400 | (136) FISGSLDRTVLLWDQR | Histone H3 (Lys4) methyltransferase complex | AT5G66240 (79/98) | *ULCS1* | A |
| Glyma.18G071300 | (105) IATASGDKTVRLWDAR | THO complex subunit 3 | AT5G56130 (88/92) | *ATTEX1* | A |
| Glyma.18G210300 | (1132) FAAGFVDGSVRLYDVR | Regulatory associated protein of mTOR | AT3G08850 (77/100) | *RAPTOR1B* | A |
| Glyma.18G284000 | (323) FATGNQDKTCRVWDVR | WD40 repeat protein | AT5G56190 (63/99) | *WD40 Family* | A |
| Glyma.19G000400 | (116) FASGSLDTNLKIWDIR | Microtubule severing protein katanin p80 subunit B | AT5G23430 (83/68) | *WD40 Family* | K |
| Glyma.19G005200 | (164) IVSGSRDGSFRIWDLR  (234) ATAGAVDSVLKFWDTR | U5 snRNP-specific protein-like factor | AT3G27640 (53/90) | *WD40 Family* | M |
| Glyma.19G029400 | (382) LLSASYDGKVMLWDLR  (336) IAAGGSDPVIRIWDPR | Microtubule binding protein YTM1 | AT5G15550 (70/99) | *ATPEP2* | M |
| Glyma.19G063300 | (131) FMSGSLDHSVRIWDLR | Histone H3 (Lys4) methyltransferase complex | AT5G14530 (85/94) | *WD40 Family* | A |
| Glyma.19G114900 | (197) IFTGGIDNDVKIWDLR  (156) VVSGSDDGTAKLWDMR  (239) LLTNGMDCKLCIWDMR | U5 snRNP-specific protein-like factor | AT2G43770 (85/100) | *WD40 Family* | T |
| Glyma.19G169500 | (358) LLSRSFDGSLKVWDLR | Uncharacterized conserved protein | AT2G20330 (69/99) | *WD40 Family* | A |
| Glyma.19G210500 | (94) LCSCSSDGTIRAWDIR | WD40 repeat protein | AT2G47790 (68/96) | *GTS1* | A |
| Glyma.19G241300 | (233) LVSGGTEKVVRVWDAR | Conserved WD40 repeat-containing protein | AT3G05090 (70/100) | *LRS1* | A |
| Glyma.19G241800 | (250) LVSGGTEKVVRVWDAR | Conserved WD40 repeat-containing protein | AT3G05090 (71/97) | *LRS1* | A |
| Glyma.20G084500 | (371) LGTGTTESLVKIWDVK | Pre-mRNA-processing factor 19 | AT2G33340 (76/99) | *MAC3B* | A |
| Glyma.20G126400 | (203) FITVSSDR KGIIYDGK | WD40 repeat stress protein/actin interacting protein | AT3G18060 (70/99) | *WD40 Family* | A |
| Glyma.20G131200 | (616) FATSSFDHDVKMWDLR | Peroxisomal targeting signal type 2 receptor | AT4G34280 (53/99) | *DHU1* | F |
| Glyma.20G132800 | (122) LITGSWDKTLKCWDPR | Cell cycle arrest protein BUB3 | AT3G19590 (82/96) | *BUB3.1* | A |
| Glyma.20G133400 | (113) LITGSWDKTLKCWDPR | Cell cycle arrest protein BUB3 | AT3G19590 (85/100) | *BUB3.1* | A |
| Glyma.20G190200 | (295) MATAGKDKKIKLWDLR | WD40 repeat protein | AT3G10530 (68/98) | *WD40 Family* | C |

^a^ The number indicates the position of each DWD motif in protein.

^b^ The numbers indicate the identity and coverage between soybean DWD protein and its corresponding homolog in Arabidopsis, respectively.

^c^ Each type is indicated in Fig 1. The Arabidopsis DWD proteins, which have been experimentally validated to interact with DDB complex, are highlighted with red color
