# Supplementary material for: Genome-wide analysis of DWD proteins in soybean (Glycine max): Significance of Gm08DWD and GmMYB176 interaction in isoflavonoid biosynthesis
Source: PLoS One. 2017 Jun 6;12(6):e0178947. doi: 10.1371/journal.pone.0178947 (PMC5460815; doi:10.1371/journal.pone.0178947)
Supplement: S3 Table — (DOCX) [file pone.0178947.s004.docx]

**Table S3** List of soybean DWD proteins which homologs are not identified as DWD in Arabidopsis by Lee et al.

| Gene ID | DWD Motif^a^ | Gene Annotation | Homolog in Arabidopsis^b^ | Gene | Type^c^ |
| --- | --- | --- | --- | --- | --- |
| Glyma.01G001600 | (489) MI SAGYEGDIKVWDFK | U3 small nucleolar RNA-associated protein 21 | AT4G04940 (72/99) | *WD40 Family* | A |
| Glyma.02G152200 | (262) ILSKSVDNEIILWEPK | Transcriptional repressor EED/ESC/FIE | AT3G20740 (78/76) | *FIE* | A |
| Glyma.02G190600 | (460) LATGGEDNTCRIWDLR  (418) AASCGLDSLARVWDLR | U4/U6 small nuclear ribonucleoprotein Prp4 | AT2G41500 (67/87) | *LIS* | Q |
| Glyma.04G129500 | (233) LLTNSNDRIIRIYENR | WD40 repeat protein | AT3G21060 (71/97) | *RBL* | A |
| Glyma.04G201300 | (361) VLSVGADRRIFGYDVR | U5 snRNP-specific protein-like factor | AT5G50970 (63/97) | *WD40 Family* | A |
| Glyma.05G010900 | (161) ILSSCTDMGGVRLWDVR | Serine/threonine kinase receptor-associated protein | AT3G15610 (82/97) | *WD40 Family* | A |
| Glyma.05G090500 | (260) LVTLSKDGNLHVWETR  (1039) MVPKGPEWGGGNWEIK | WD40 repeat protein | AT3G50590 (68/100) | *WD40 Family* | M |
| Glyma.06G163900 | (380) VLSVGADRRIFGYDVR | U5 snRNP-specific protein-like factor | AT5G50970 (61/97) | *WD40 Family* | A |
| Glyma.06G185900 | (296) VYSGACDRAILVWEKK | Microtubule binding protein YTM1 | AT5G50120 (55/90) | *WD40 Family* | A |
| Glyma.07G248900 | (138) IVSGSFDETVRVWDVK | WD40 repeat protein | AT3G49660 (74/98) | *ATWDR5A* | A |
| Glyma.07G273800 | (162) QLWAGQEGGVRVWEIK | Inositol polyphosphate 5-phosphatase | AT2G43900 (69/98) | *5PTASE12* | H |
| Glyma.08G337200 | (331) LVSASQDGTIKIWEDR | Enhancer of mRNA-decapping protein 4 | AT3G13300 (57/96) | *VCS* | A |
| Glyma.09G016800 | (417) LASA SADGTVKIWEER | Nuclear protein | AT3G13300 (52/91) | *VCS* | A |
| Glyma.10G000800 | (460) LATGGEDNTCRIWDLR  (418) AASCGLDSLARVWDLR | U4/U6 small nuclear ribonucleoprotein PRP4 | AT2G41500 (67/87) | *LIS* | Q |
| Glyma.10G022000 | (263) ILSKSVDNEIILWEPK | Transcriptional repressor EED/ESC/FIE | AT3G20740 (78/75) | *FIE* | A |
| Glyma.10G129800 | (108) VASGDDEGCIKVWDTR | WD40 repeat protein | AT2G34260 (70/100) | *WDR55* | A |
| Glyma.10G284000 | (231) FAVAGSDEYARLYDIR | WD40 repeat protein 42A | AT4G35140 (66/89) | *WD40 Family* | A |
| Glyma.11G120700 | (82) VVSGGMDTLVCVWDLK  (256) VCVQSIEHPGCVWDAK | Phospholipase A2-activating protein | AT3G18860 (71/99) | *WD40 Family* | M |
| Glyma.12G025800 | (97) VASGDDDGCIKVWDTR | WD40 repeat protein | AT2G34260 (63/100) | *WDR55* | A |
| Glyma.12G045800 | (82) VVSGGMDTLVCVWDLK  (256) VCVQSIEHPGCVWDAK | Phospholipase A-2-activating protein | AT3G18860 (72/99) | *WD40 Family* | M |
| Glyma.13G227200 | (93) MYSGSEDGTVKIWDLR | G-protein beta subunit-like protein | AT3G18140 (87/97) | *LST8-1* | A |
| Glyma.13G287200 | (248) ILSKSVDNEIILWEPK | Transcriptional repressor EED/ESC/FIE | AT3G20740 (75/77) | *FIE* | A |
| Glyma.15G085200 | (93) MYSGSEDGTVKIWDLR | G-protein beta subunit-like protein | AT3G18140 (87/97) | *LST8-1* | A |
| Glyma.15G122400 | (316) LASASADGTVKIWEER | Nuclear protein | AT3G13300 (53/99) | *VCS* | A |
| Glyma.15G155900 | (360) FLASSMDGSMRLYDLR | WD40 repeat protein 21A | AT5G17370 (43/98) | *WD40 Family* | A |
| Glyma.17G025400 | (140) IVSGSFDETVRVWDVK | WD40 repeat protein | AT3G49660 (74/93) | *ATWDR5A* | A |
| Glyma.17G176300 | (260) LITLSKDGNLHVWETR  (1017) MVPKGPEWGGGNWEIK | WD40 repeat protein | AT3G50590 (68/100) | *WD40 Family* | N |
| Glyma.18G072400 | (135) LVSASQDGTIKIWEDR | Nuclear protein | AT3G13300 (58/100) | *VCS* | A |
| Glyma.19G020400 | (196) LIRTGGDGRAMLWDVR | G-protein beta subunit-like protein | AT5G25150 (70/98) | *TAF5* | A |
| Glyma.20G105700 | (205) FAVAGS DEYARLYDIR | WD repeat-containing protein 42A | AT4G35140 (65/94) | *WD40 Family* | A |
| Glyma.20G173100 | (331) VASGCVDGKVRLWDSR | Angio-associated migratory cell protein | AT1G71840 (63/97) | *WD40 Family* | A |

^a^ The number indicates the position of each DWD motif in protein.

^b^ The numbers indicate the identity and coverage between soybean DWD protein and its corresponding homolog in Arabidopsis, respectively.

^c^ Each type is indicated in Fig 1. The Arabidopsis DWD proteins, which have been experimentally validated to interact with DDB complex, are highlighted with red color
